# Characterization of baseline polybacterial versus monobacterial infections in three randomized controlled bacterial conjunctivitis trials and microbial outcomes with besifloxacin ophthalmic suspension 0.6%

**DOI:** 10.1371/journal.pone.0237603

**Published:** 2020-08-25

**Authors:** Heleen H. DeCory, Christine M. Sanfilippo, Howard M. Proskin, Joseph M. Blondeau

**Affiliations:** 1 Medical Affairs, Bausch + Lomb, Rochester, New York, United States of America; 2 Howard M. Proskin & Associates, Rochester, New York, United States of America; 3 Clinical Microbiology, Royal University Hospital, Saskatoon, Saskatchewan, Canada; University of Pretoria, SOUTH AFRICA

## Abstract

**Background/Purpose:**

To date, studies examining polymicrobial infections in ocular disease have mostly been limited to keratitis or endophthalmitis. We characterized polybacterial infections compared to monobacterial infections in prior clinical studies evaluating besifloxacin ophthalmic suspension 0.6% for the treatment of bacterial conjunctivitis and report on associated microbiological outcomes.

**Methods:**

In this post-hoc analysis, microbiological data for subjects with conjunctivitis due to one or more than one bacterial species in three previous studies (two vehicle-, one active-controlled) of besifloxacin were extracted. Bacterial species identified at baseline were deemed causative if their colony count equaled or exceeded species-specific prespecified threshold criteria. In subjects with polybacterial infections, the fold-increase over threshold was used to rank order the contribution of individual species. Baseline pathogens and their minimum inhibitory concentrations (MICs) for common ophthalmic antibiotics were compared by infection type, as were microbial eradication rates following treatment with besifloxacin.

**Results:**

Of 1041 subjects with culture-confirmed conjunctivitis, 17% had polybacterial and 83% had monobacterial conjunctivitis at baseline. In polybacterial compared to monobacterial infections, *Haemophilus influenzae* and *Streptococcus pneumoniae* were identified less frequently as the dominant infecting species (*P* = 0.042 and *P*<0.001, respectively), whereas *Streptococcus mitis*/S. mitis group was identified more frequently as dominant (*P*<0.001). Viral coinfection was also identified more frequently in polybacterial infections (*P*<0.001). *Staphylococcus aureus* was the most common coinfecting species in polybacterial infections and the second most common dominant species in such infections. With few exceptions, MICs for individual species were comparable regardless of infection type. Clinical microbial eradication rates with besifloxacin were high regardless of infection type (*P*≤0.016 vs vehicle at follow-up visits).

**Conclusions:**

Approximately one in five subjects with bacterial conjunctivitis are infected with more than one bacterial species underscoring the need for a broad-spectrum antibiotic for such infections. Besifloxacin treatment resulted in robust eradication rates of these infections comparable to monobacterial infections.

**Trial registration:**

NCT000622908, NCT00347932, NCT00348348

## Introduction

Acute bacterial conjunctivitis is a common eye infection treated by primary care practitioners and pediatricians and one of the most commonly encountered eye problems in medicine [1,2]. Characterized most often by redness and mild-to-moderate purulent conjunctival discharge as well as by crusting and sticking or gluing of the eyelids upon waking, the disease is generally self-limited [3,4]. However, treatment with a topical ocular anti-infective shortens the duration of the disease, reduces the risk of complications, and reduces contagious spread, which is important especially for children, allowing them to return to school and/or day care faster [3].

Current treatment of bacterial conjunctivitis is empiric, based upon the likely causative bacterial pathogens, which include staphylococcal species (*S*. *aureus* and coagulase-negative staphylococci [CoNS], common in adults and adolescents), *Streptococcus pneumoniae*, *Haemophilus influenzae* (most common among children), *Moraxella catarrhalis*, and *Pseudomonas aeruginosa* (most common in contact lens wearers) [1,4]. These bacteria may spread via hand-to-eye contact or through colonization from adjacent tissues such as the nasal or sinus mucosa. Advances in diagnostics have led to a growing recognition of the polymicrobial nature of many infections [5–9], including topical ocular infections [10–14]. Moreover, research suggests that interactions between multiple bacterial species including commensal bacteria or other microbes (eg, viruses, fungi) may impact disease progression and treatment success. For instance, studies of polymicrobial infections have documented alterations in virulence factors, biofilm formation, and antibiotic resistance or tolerance [5]. While much research has focused on the pathogenesis of the polymicrobial nature of systemic infections, research on polymicrobial infections of the eye has been limited to studies reporting on the frequency of such infections in keratitis and/or endophthalmitis, with rates ranging from 2–50% [10,11,14,15–23], or as high as 83% for endophthalmitis using polymerase chain reaction amplification techniques [24].

Besivance^®^ (besifloxacin ophthalmic suspension, 0.6%; Bausch & Lomb Incorporated; Rochester, NY) was approved in 2009 by the United States Food and Drug Administration for the treatment of bacterial conjunctivitis. A chlorinated fluoroquinolone, besifloxacin has potent activity against both Gram-positive and Gram-negative bacteria, including multidrug-resistant strains [25–27], and is rapidly bactericidal [28–30]. Besifloxacin is formulated with DuraSite^®^ (Sun Pharma Global FZE), which helps increase retention on the ocular surface, and has demonstrated effectiveness in several bacterial conjunctivitis clinical trials [31–36].

To gain insight into the contribution of polymicrobial infections to ocular surface disease, we assessed the frequency of such infections in three prior clinical pivotal studies of besifloxacin ophthalmic suspension 0.6% in the treatment of bacterial conjunctivitis. This paper reports on characteristics of baseline polybacterial infections in these studies in comparison to monobacterial infections, including baseline minimum inhibitory concentrations to common ophthalmic antibiotics. We also present microbial eradication outcomes in subjects with such infections following treatment with besifloxacin.

## Materials and methods

This was a post-hoc analysis of microbiological data from three similarly designed prospective, randomized, multicenter, double-masked clinical trials (two vehicle-controlled and one active [moxifloxacin]-controlled; ClinicalTrials.gov identifiers: NCT00347932, NCT00348348, NCT00622908) evaluating besifloxacin ophthalmic suspension, 0.6% in the treatment of bacterial conjunctivitis. All three studies were conducted in accordance with Good Clinical Practices, the International Conference on Harmonization guidelines, the Declaration of Helsinki, and the Health Insurance Portability and Accountability Act guidelines. All three studies were approved by the Institutional Review Board of each study center or by Schulman Associates Institutional Review Board, Inc. (Cincinnati, OH) or Western Institutional Review Board Incorporated (Olympia, WA) when a local review board was not available. Prior to enrollment in the study, all patients (or legally authorized representatives for patients less than 18 years of age) gave written informed consent. Details of clinical study designs and results of individual studies have been reported [31–33].

In all studies, subjects aged ≥1 year with bacterial conjunctivitis, as evidenced by grade 1 (ie, mild) or greater purulent conjunctival discharge and bulbar conjunctival injection in at least one eye, were eligible to participate. Additional inclusion and exclusion criteria have been described [37]. Patients completed three study visits. At the first visit (day 1), eligibility was determined by: a clinical assessment of ocular signs and symptoms in both eyes; an eye examination that included pinhole visual acuity, biomicroscopy, and ophthalmoscopy; and culture of the infected eye(s). Separate bacterial and viral cultures were taken from the cul-de-sac of the affected eye(s) prior to instillation of any medication, and samples were shipped refrigerated or frozen (in 20% glycerol for bacteria and M4RT viral transport media for viruses) to Covance Central Laboratory Services, Inc. (Indianapolis, IN) for quantitative and qualitative microbiology to enumerate and identify baseline bacterial pathogens and to identify the presence of any coinfecting virus or yeast as previously described [27]. To avoid the attribution of the infection to commensal (ie, normal) microflora, pathogens were identified as causative only if their colony count (CFU/mL) equaled or exceeded prespecified species-specific threshold criteria based on the Cagle list as modified by Leibowitz (Table 1) [38,39].

**Table 1:** Species-specific prespecified threshold criteria (adapted from Leibowitz).

Group [Threshold criteria]	Species
I [1 CFU/mL]	Streptococcus pyogenes Streptococcus pneumoniae Gram-negative spp. (except *Moraxella catarrhalis*)
II [10 CFU/mL]	Staphylococcus aureus *Streptococcus* spp. (except *S*. *pyogenes* and *S*. *pneumoniae*) Moraxella catarrhalis
III [100 CFU/mL]	*Staphylococcus* spp. (except *S*. *aureus*) *Micrococcus* spp. *Bacillus* spp.
IV [1000 CFU/mL]	*Corynebacterium* spp.

Following culture of infected eye(s), subjects self-administered one drop of study medication three times daily (TID) at approximately 6-hour intervals for 5 days. Clinical assessments performed at visit 1 and cultures from the cul-de-sac were repeated at visit 2 (day 4 [± 1] [33] or day 5 [± 1] [31,32]) and visit 3 (day 8 or 9) [31–33]. Microbial eradication of the baseline bacterial infection (binary outcome) was defined as the absence of all ocular bacterial species that were present at or above threshold at baseline (visit 1) in the study eye, defined as the eye with the highest combined score of conjunctival discharge and/or bulbar conjunctival injection at baseline.

In each study, minimum inhibitory concentrations (MICs) for besifloxacin and comparator antibacterial agents (including other fluoroquinolones) were determined for all bacterial isolates at or above threshold at baseline (visit 1) by broth microdilution according to the procedure recommended by the Clinical and Laboratory Standards Institute (CLSI) [27]. As well, *S*. *aureus* and *S*. *epidermidis* MICs for oxacillin were interpreted according to the susceptibility/resistance criteria published by CLSI [27] in order to characterize isolates by methicillin resistance phenotype.

For the post-hoc analysis, subjects with only one bacterial species in the study eye above prespecified threshold criteria (ie, monobacterial conjunctivitis) or more than one bacterial species in the study eye above prespecified threshold criteria (ie, polybacterial conjunctivitis) at baseline were identified across the three studies. Because study designs, assessments, and endpoints were the same across the three studies, and culture collection and laboratory analysis procedures (conducted at the same central laboratory) were the same across the three studies, subject demographics and baseline microbiological (ie bacterial and viral infection) data for subjects with monobacterial and polybacterial infections were extracted and pooled for an integrated analysis. Individual bacterial species at or above threshold in subjects with cultured-confirmed monobacterial or polybacterial conjunctivitis were tabulated along with their minimum inhibitory concentrations and antimicrobial resistance phenotypes, where indicated. In addition, the prevalence of a polysaccharide capsule among *S*. *pneumoniae* isolates was evaluated through cross-tabulation against data from a prior analysis evaluating capsule presence using antisera [40]. To evaluate the relative contribution of each bacterial species in each polybacterial infection, the fold-increase in colony count (CFU/mL) over the prespecified threshold (CFU/mL) was used to rank order the contribution of each causative bacterial species. Microbial eradication outcomes for subjects with monobacterial or polybacterial conjunctivitis treated with besifloxacin, vehicle, or active comparator (moxifloxacin) were also extracted and pooled at each follow-up visit for an integrated analysis of clinical treatment outcomes. In the analyses of microbial eradication outcomes, missing data were imputed as failures.

### Statistical methods

A two-way analysis of variance (ANOVA) with fixed effects for infection type (ie, monobacterial and polybacterial) and clinical study was employed to evaluate any difference in age between subjects presenting with monobacterial and polybacterial conjunctivitis. Cochrane-Mantel Haenszel (CMH) tests stratified by clinical study were employed for the comparison of subjects’ gender and presence of viral con-infection. Fisher’s exact tests were performed for the comparison between infection types with respect to frequency of dominant infecting species, and the comparison of the proportion of encapsulated *S*. *pneumoniae* organisms. Fisher’s exact tests were also performed to compare the proportions of methicillin-resistant *S*. *aureus* an *S*. *epidermidis* isolates.

Pairwise comparisons between treatments of microbial eradication were performed using chi-squared tests. Preliminary chi-squared tests were performed for each treatment at each visit and each study to confirm the nominal consistency of microbial eradication across the three clinical studies.

All statistical tests of hypothesis employed a level of significance of α = 0.05.

## Results

### Study population and pathogen distribution at baseline

**Fig 1:**
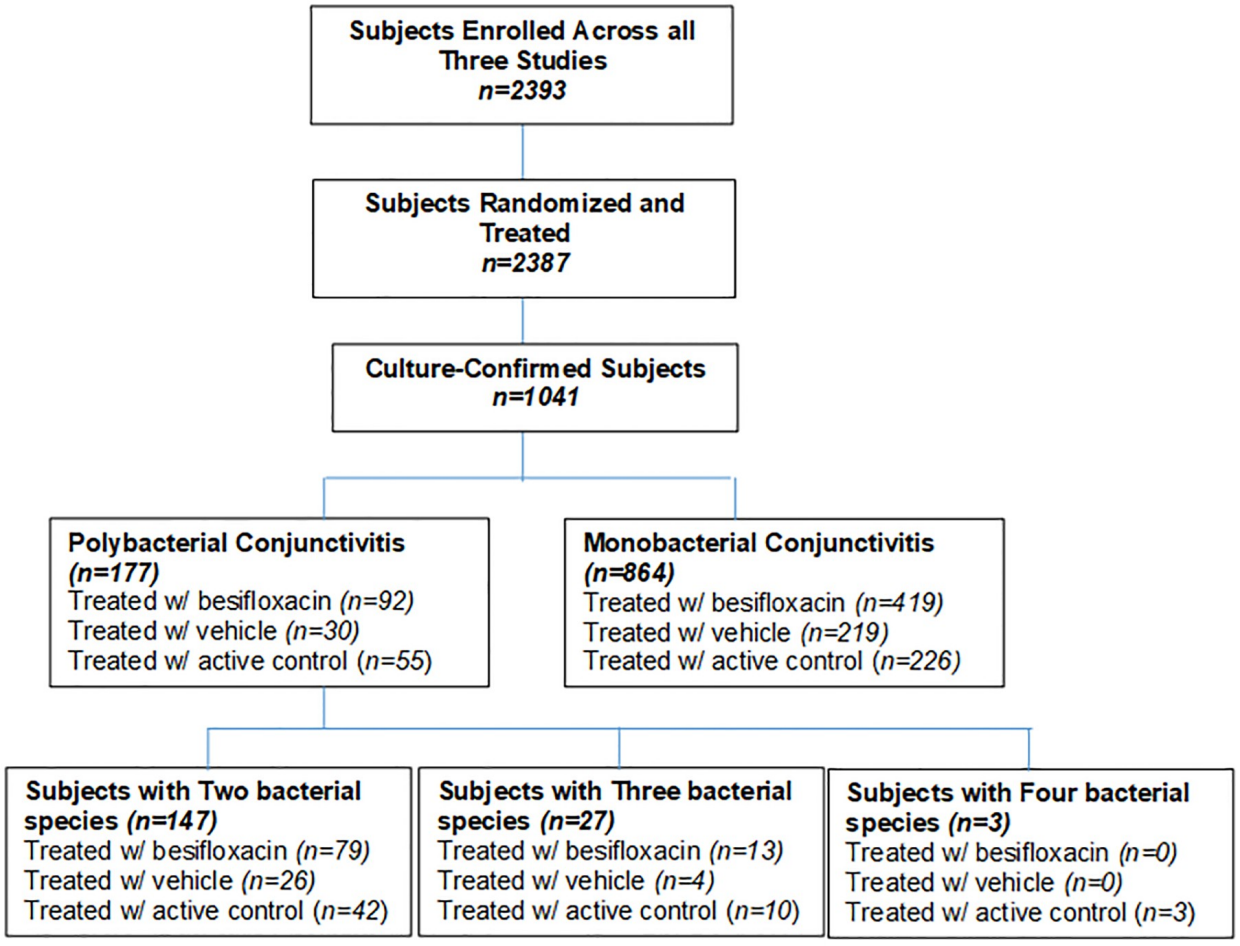
Distribution of subjects.

Subject distribution is shown in Fig 1. Of 2393 subjects enrolled across all three studies, 2387 were randomized and treated, and of these 1041 (43.6%) had culture-confirmed bacterial conjunctivitis in the study eye. The majority, or 864 (83%) of subjects with culture-confirmed bacterial conjunctivitis, were infected with only one bacterial species above the prespecified species-specific threshold criteria and were thus classified as having monobacterial conjunctivitis, while 177 (17%) of subjects were infected with more than one bacterial species above species-specific threshold criteria and were thus classified as having polybacterial conjunctivitis. Among subjects with polybacterial conjunctivitis infections, 147 (83%) were infected with two bacterial species, 27 (15%) with three species, and 3 (2%) with four species.

**Fig 2:**
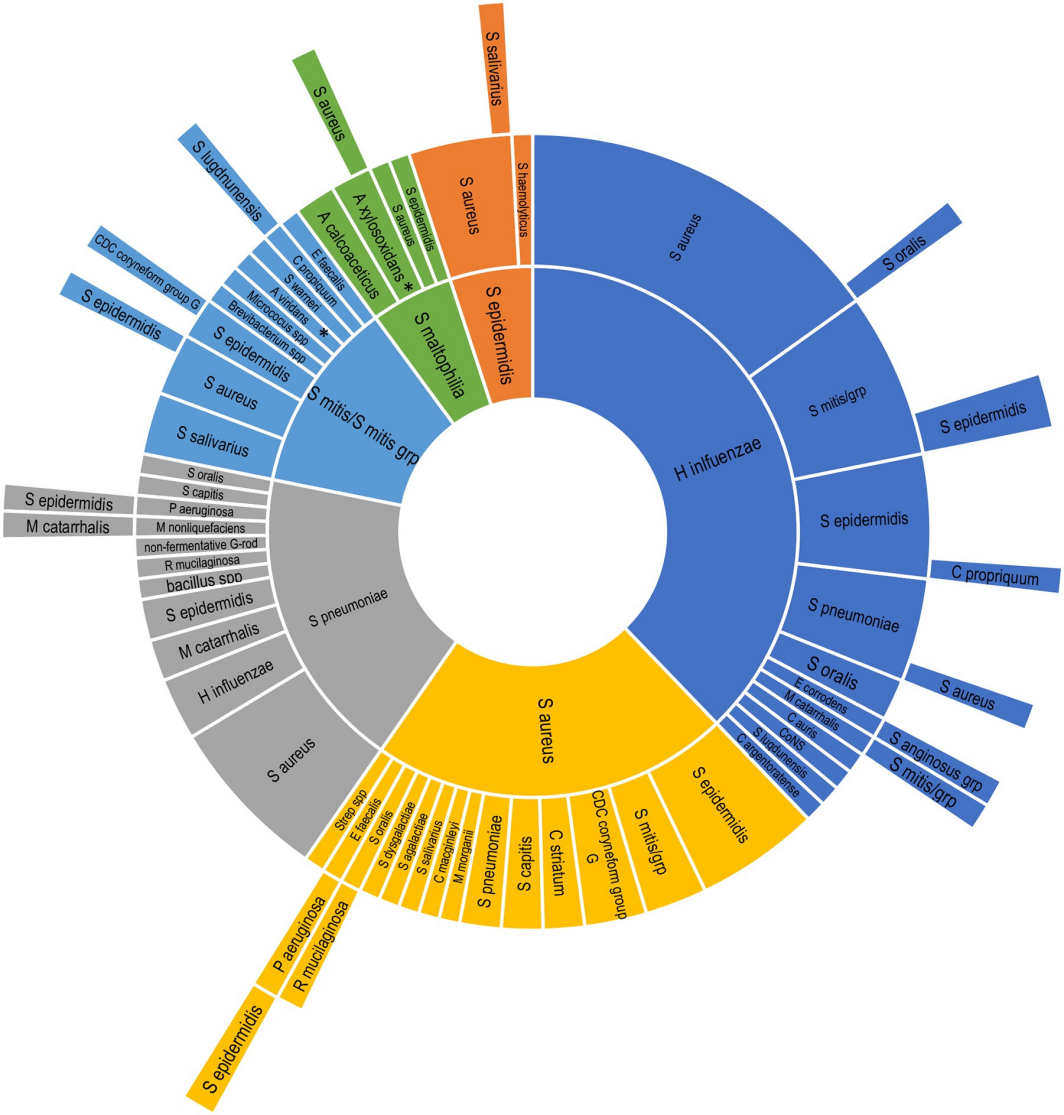
Dominant, secondary, tertiary, and quaternary infecting species at baseline in polybacterial conjunctivitis infections. Dominant bacterial species are shown in the inner ring, whereas secondary, tertiary, and quaternary infecting bacterial species are shown by rank order moving outwards by ring. Only those polybacterial infections in which the same dominant species was identified in more than 5 infections are presented. Co-dominant species are indicated with an asterisk.

While several isolates from both polybacterial and monobacterial infections were only identified at the genus or group level, 65 and 58 different bacterial species, respectively, were identified above threshold among polybacterial and monobacterial infections. Table 2 summarizes baseline pathogens with a frequency >2.0% among either poly- or monobacterial infections. The four most common species identified among both poly- and monobacterial infections were *S*. *aureus*, *H*. *influenzae*, *S*. *epidermidis*, and *S*. *pneumoniae*, together accounting for 50.1% (194/387) and 83.8% (724/864) of isolates above threshold in poly- and monobacterial infections, respectively. Fig 2 presents the distribution of dominant, secondary, tertiary, and quaternary infecting bacterial species in subjects with polybacterial infections at baseline. *H*. *influenzae* was the most common dominant infecting bacterial species (25.4% of infections), and *S*. *aureus* the second most common (14.7% of infections). *S*. *aureus* was also the most common secondary coinfecting species in polybacterial infections (25.4% of infections), while *S*. *epidermidis* was the second most common secondary coinfecting bacterial species (15.3% of infections).

**Table 2:** Baseline pathogens at a frequency of >2.0%* in poly- and monobacterial conjunctivitis infections.

Organism	Polybacterial (N° of isolates = 387; N° of subjects = 177)		Monobacterial (N° of isolates = 864; N° of subjects = 864)	
	n	% isolates (*% subjects*)	n	% isolates/ subjects
*Staphylococcus aureus*	73	18.9 (*41*.*2*)	110	12.7
*Haemophilus influenzae*	51	13.2 (*28*.*8*)	288	33.3
*Staphylococcus epidermidis*	41	10.6 (*23*.*2*)	54	6.2
*Streptococcus pneumoniae*	29	7.5 (*16*.*4*)	272	31.5
Streptococcus mitis group	24	6.2 (*13*.*6*)	17	2.0
*Streptococcus oralis*	14	3.6 (*7*.*9*)	3	0.3
CDC Coryneform Group G	10	2.6 (*5*.*6*)	13	1.5
*Streptococcus mitis*	10	2.6 (*5*.*6*)	4	0.5
*Moraxella catarrhalis*	7	1.8 (*4*.*0*)	5	0.6
*Stenotrophomonas maltophilia*	7	1.8 (*4*.*0*)	2	0.2
*Streptococcus salivarius*	7	1.8 (*4*.*0*)	0	—
Streptococcus species	7	1.8 (*4*.*0*)	5	0.6
*Aerococcus viridans*	5	1.3 (*2*.*8*)	3	0.3
*Corynebacterium* macginleyi	5	1.3 (*2*.*8*)	1	0.1
*Corynebacterium striatum*	5	1.3 (*2*.*8*)	3	0.3
*Pseudomonas aeruginosa*	5	1.3 (*2*.*8*)	3	0.3
*Serratia marcescens*	5	1.3 (*2*.*8*)	4	0.5
*Corynebacterium propinquum*	4	1.0 (*2*.*3*)	2	0.2
*Corynebacterium pseudodiphtheriticum*	4	1.0 (*2*.*3*)	3	0.3
*Staphylococcus lugdunensis*	4	1.0 (*2*.*3*)	2	0.2
Other species at a frequency of ≤2.0%	70	18.1 (*39*.*5*)	70	8.1

*The complete listing for the frequency of all baseline pathogens for all subjects can be found in S1 File.

Respective summaries of demographics for subjects with polybacterial conjunctivitis and for subjects with monobacterial conjunctivitis are presented and compared in Table 3. Additionally, Table 3 presents the most frequent dominant causative infecting bacterial species in polybacterial infections compared with the corresponding frequency of those species in monobacterial infections. There were no differences among subjects with poly- and monobacterial conjunctivitis infections with respect to demographics. However, a greater proportion of subjects with polybacterial infections had concurrent viral infection or presented with *S*. *mitis*/S. mitis group spp, *S*. *oralis*, and *Stenotrophomonas maltophilia* as the dominant infecting species as compared to subjects with monobacterial infections. In contrast, a smaller proportion of subjects with polybacterial infections presented with *H*. *influenzae* or *S*. *pneumoniae* as the dominant infecting species as compared to subjects with monobacterial infections. Further analysis of encapsulation data for *S*. *pneumoniae* isolates showed that a significantly greater proportion of the *S*. *pneumoniae* isolates from polybacterial infections were encapsulated compared to those from monobacterial infections (27.3% [6/22] vs 4.8% [13/272], *P*<0.001). No subjects with either polybacterial or monobacterial conjunctivitis infections at baseline were concurrently infected with fungal pathogens.

**Table 3:** Subject demographics and baseline pathogens in poly- and monobacterial conjunctivitis infections.

	All culture-confirmed subjects (N = 1,041)				
	Polybacterial Infections (n = 177)		Monobacterial Infections (n = 864)		*P*-value
Age, years					
mean (SD)	32.9	(28.9)	29.7	(25.3)	0.330^a^
Min, max	1,	98	0,	100	
Gender, female n (%)	100	(56.5)	488	(56.5)	0.862^b^
Viral coinfection, n (%)^c^	14	(7.9)	14	(1.6)	<0.001^b^
Dominant infecting species, n (%)					
*H*. *influenzae*	45	(25.4)	288	(33.3)	0.042^d^
*S*. *aureus*	26	(14.7)	110	(12.7)	0.465^d^
*S*. *pneumoniae*	22	(12.4)	272	(31.5)	<0.001^d^
*encapsulated*	6	(3.4)	13	(1.5)	
*S*. *mitis*/S. mitis group	14	(7.9)	21	(2.4)	<0.001^d^
*S*. *epidermidis*	6	(3.4)	54	(6.2)	0.158^d^
*S*. *maltophilia*	6	(3.4)	2	(0.2)	<0.001^d^
*S*. *oralis*	5	(2.8)	3	(0.3)	0.005^d^

^a^ANOVA with fixed effects of infection type and clinical study

^b^CMH tests stratified by clinical study.

^c^Twelve subjects in each group were infected with adenovirus and two with herpes simplex virus.

^d^Fisher’s exact tests.

Only those species identified as dominant ≥ 5 times among polybacterial infections are shown.

Analyses and corresponding p-values can be found in S1 File.

### Antibiotic minimum inhibitory concentrations

Table 4 presents MIC ranges and MIC_90_s of besifloxacin and comparator antibiotics for all isolates, Gram-positive isolates, Gram-negative isolates, and for individual species from both mono- and polymicrobial conjunctivitis infections with ≥10 isolates at baseline in either of the groups. Overall, the MIC that inhibited 90% of all above-threshold isolates from mono-/polybacterial infections was 0.125/0.5 μg/mL for besifloxacin, 0.25/1 μg/mL for moxifloxacin, 0.5/1 μg/mL for gatifloxacin, 1/2 μg/mL for levofloxacin, 1/4 μg/mL for ciprofloxacin and 2/2 μg/mL for ofloxacin, or within one to two dilution differences from one another.

**Table 4:** Minimum inhibitory concentrations of besifloxacin and comparator antibacterial agents for baseline isolates from poly- and monobacterial conjunctivitis infections.

	MIC (μg/mL	Besi	Moxi	Gati	Levo	Cipro	Oflox	Azi
**Polybacterial Infections**								
All isolates (n = 387)	Range	0.015–8	0.008–>8	0.008–>8	0.008–>8	0.008–>8	0.008–>8	0.008–>8
	MIC_90_	0.5	1	1	2	4	2	>8
Gram-positive isolates (n = 286)	Range	0.015–4	0.008–>8	0.008–>8	0.008–>8	0.015–>8	0.008–>8	0.008–>8
	MIC_90_	0.5	2	2	4	8	8	>8
Gram-negative isolates (n = 101)	Range	0.015–8	0.015–8	0.008–8	0.008–8	0.015->8	0.03->8	0.015->8
	MIC_90_	2	1	1	1	1	2	>8
*S*. *aureus* (n = 73)	Range	0.015–4	0.03–>8	0.03–>8	0.03–>8	0.06–>8	0.125–>8	0.06–>8
	MIC_90_	0.5	2	2	4	>8	8	>8
*H*. *influenzae* (n = 51)	Range	0.015–0.5	0.015–1	0.008–0.5	0.008–1	0.008–1	0.03–2	0.015–4
	MIC_90_	0.06	0.06	0.03	0.03	0.03	0.06	2
*S*. *epidermidis* (n = 41)	Range	0.03–4	0.06–>8	0.125–>8	0.25–>8	0.125–>8	0.5–>8	0.5–>8
	MIC_90_	0.5	4	4	8	>8	>8	>8
*S*. *mitis/*S. mitis group (n = 34)	Range	0.03–1	0.06–2	0.25–2	0.5->8	0.5->8	1->8	0.03->8
	MIC_90_	0.25	0.25	1	2	4	4	8
*S*. *pneumoniae* (n = 29)	Range	0.06–0.25	0.06–1	0.125–0.5	0.5–2	0.25–2	1–4	0.125->8
	MIC_90_	0.125	0.25	0.5	1	2	2	>8
*S*. *oralis* (n = 14)	Range	0.125–0.25	0.015–0.5	0.03–1	0.125–2	0.03–4	0.125–4	0.0.06–>8
	MIC_90_	0.25	0.25	1	2	4	4	>8
*CDC* Coryneform Group G (n = 10)	Range	0.015–2	0.06->8	0.03–8	0.06->8	0.03–8	0.125->8	0.06->8
	MIC_90_	2	>8	8	>8	8	>8	>8
**Monobacterial Infections**								
All isolates (n = 864)	Range	0.008–8.0	≤0.004->8	≤0.004->8	≤0.004->8	≤0.004->8	0.015->8	0.015->8
	MIC_90_	0.125	0.25	0.5	1	1	2	>8
Gram-positive isolates (n = 537)	Range	0.008–8.0	0.015->8	0.015->8	0.03->8	0.015->8	0.06->8	0.015->8
	MIC_90_	0.125	0.25	0.5	1	2	2	>8
Gram-negative isolates (n-327)	Range	0.008–8.0	≤0.004–4	≤0.004–4	≤0.004–4	≤0.004->8	0.015–8	0.015->8
	MIC_90_	0.125	0.125	0.125	0.125	0.06	0.25	4
*S*. *aureus* (n = 110)	Range	0.008–8.0	0.03>8	0.03->8	0.06->8	0.06->8	0.125->8	1->8
	MIC_90_	1	2	4	8	>8	>8	>8
*H*. *influenzae* (n = 288)	Range	0.008–0.5	0.008–1	≤0.004–0.5	≤0.004–1	≤0.004–1	0.015–2	0.015->8
	MIC_90_	0.06	0.06	0.03	0.03	0.015	0.06	4
*S*. *epidermidis* (n = 54)	Range	0.03–2.0	0.06->8	0.06->8	0.125->8	0.125->8	0.25->8	0.5->8
	MIC_90_	0.5	4	2	8	>8	>8	>8
*S*. *mitis/*S. mitis group (n = 21)	Range	0.03–0.25	0.03–0.5	0.06–1	0.125–2	0.06–4	0.25–4	0.03–8
	MIC_90_	0.25	0.25	0.5	2	2	2	8
*S*. *pneumoniae* (n = 272)	Range	0.03–0.25	0.06–0.5	0.125–1	0.125–2	0.125->8	0.5–2	0.06->8
	MIC_90_	0.125	0.125	0.5	1	1	2	>8
*S*. *oralis* (n = 3)	Range	0.06–0.125	0.125–0.25	0.25–0.5	0.5–1	1–2	1–2	0.06–0.125
	MIC_90_	—	—	—	—	—	—	—
*CDC* Coryneform Group G (n = 13)	Range	0.008–0.06	0.03–0.125	0.03–0.5	0.06–1	0.03–0.5	0.125–2	0.06->8
	MIC_90_	0.06	0.25	0.5	0.5	0.25	1	>8

MIC_90_: Minimum inhibitory concentration that inhibits growth of 90% of isolates

MIC data is only shown for those baseline species with ≥10 isolates in either subgroup

Azi, azithromycin; Besi, besifloxacin; Cipro, ciprofloxacin; Gati, gatifloxacin, Levo, levofloxacin; Moxi, moxifloxacin; Oflox, Ofloxacin

Among *S*. *aureus* isolates, 13.7% (10/73) from polybacterial infections and 15.5% (17/110) from monobacterial infections were methicillin-resistant (MRSA; *P* = 0.883), while among *S*. *epidermidis* isolates, 46.3% (19/41) of those obtained from polybacterial infections and 38.9% (21/54) obtained from monobacterial infections were methicillin-resistant (MRSE; *P* = 0.532). Evaluation of MICs of besifloxacin and comparator antibiotics against MRSA and MRSE isolates showed no apparent differences between those derived from polybacterial compared to monobacterial infections. Specifically, MIC_90_s for MRSA from mono-/polybacterial infections were 4/1 μg/mL for besifloxacin, >8/8 μg/mL for moxifloxacin, and >8/>8 μg/mL for gatifloxacin, levofloxacin, ciprofloxacin, ofloxacin. Similarly, the MIC_90_s for MRSE from mono-/polybacterial infections were 0.5/4 μg/mL for besifloxacin, 2/>8 μg/mL for moxifloxacin and gatifloxacin, and 8/>8 μg/mL for levofloxacin, ciprofloxacin, and ofloxacin.

**Fig 3:**
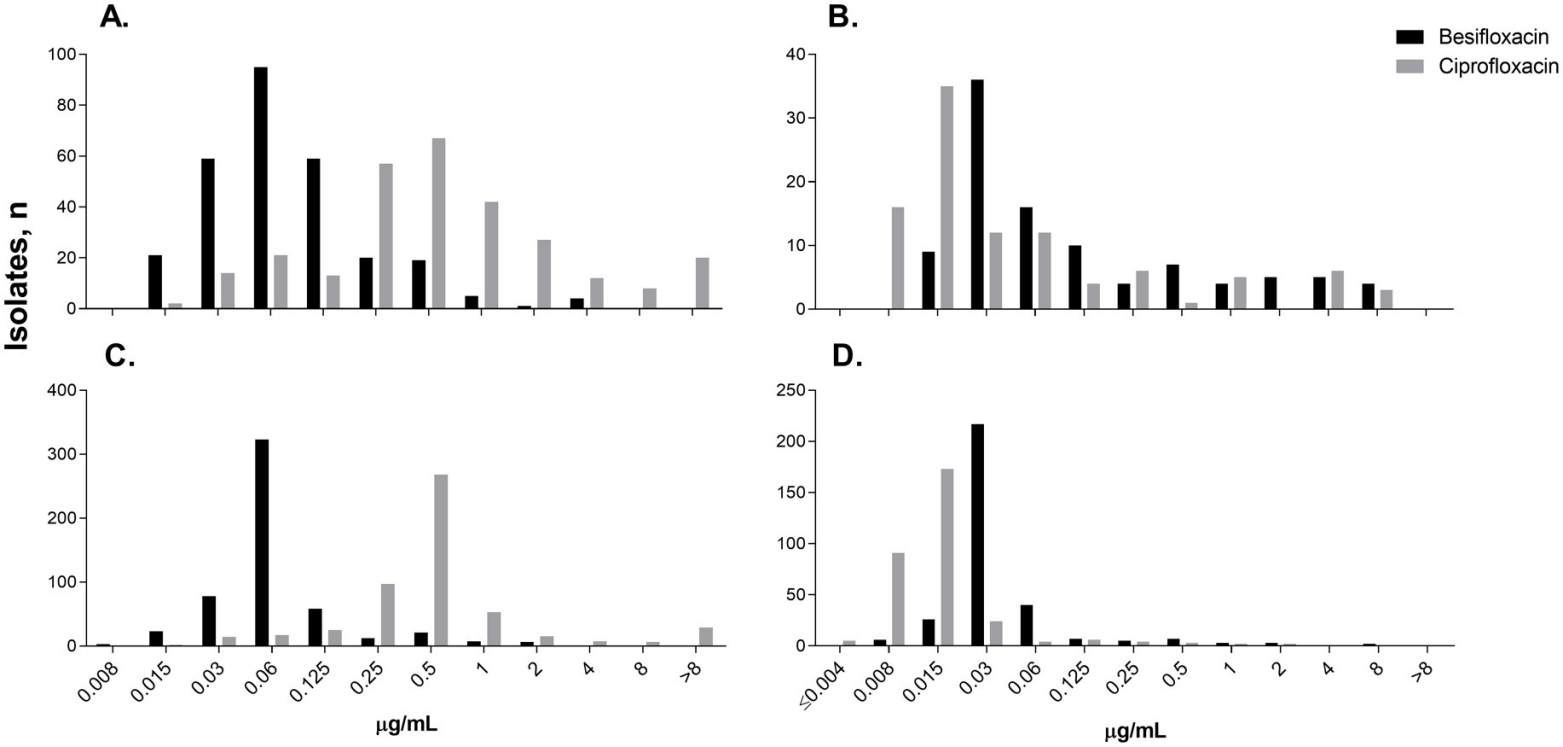
Distribution of Minimum Inhibitory Concentrations (MICs) for besifloxacin and ciprofloxacin against gram-positive and gram-negative isolates from poly- and monobacterial conjunctivitis infections. A. Gram-positive isolates from polybacterial infections (n = 286), B. Gram-negative isolates from polybacterial infections (n = 101), C. Gram-positive isolates from monobacterial infections (n = 537), and D. Gram-negative isolates from monobacterial infections (n = 327).

Fig 3 presents the distribution of MICs for besifloxacin compared to ciprofloxacin against Gram-positive and Gram-negative isolates from poly- and monobacterial infections. For mono/polybacterial infections, respectively, corresponding MIC_90_s were 0.125/0.5 μg/mL for besifloxacin and 2/8 μg/mL for ciprofloxacin for Gram-positive isolates and 0.125/2 μg/mL for besifloxacin and 0.06/1 μg/mL for ciprofloxacin for Gram-negative isolates (Table 4).

### Microbial eradication rates

The preliminary chi-squared tests performed for each treatment at each visit indicated no statistically significant difference among the within-treatment microbial eradication rates across the three clinical studies. Subsequent analyses on eradication rates were performed utilizing the pooled study data.

**Fig 4:**
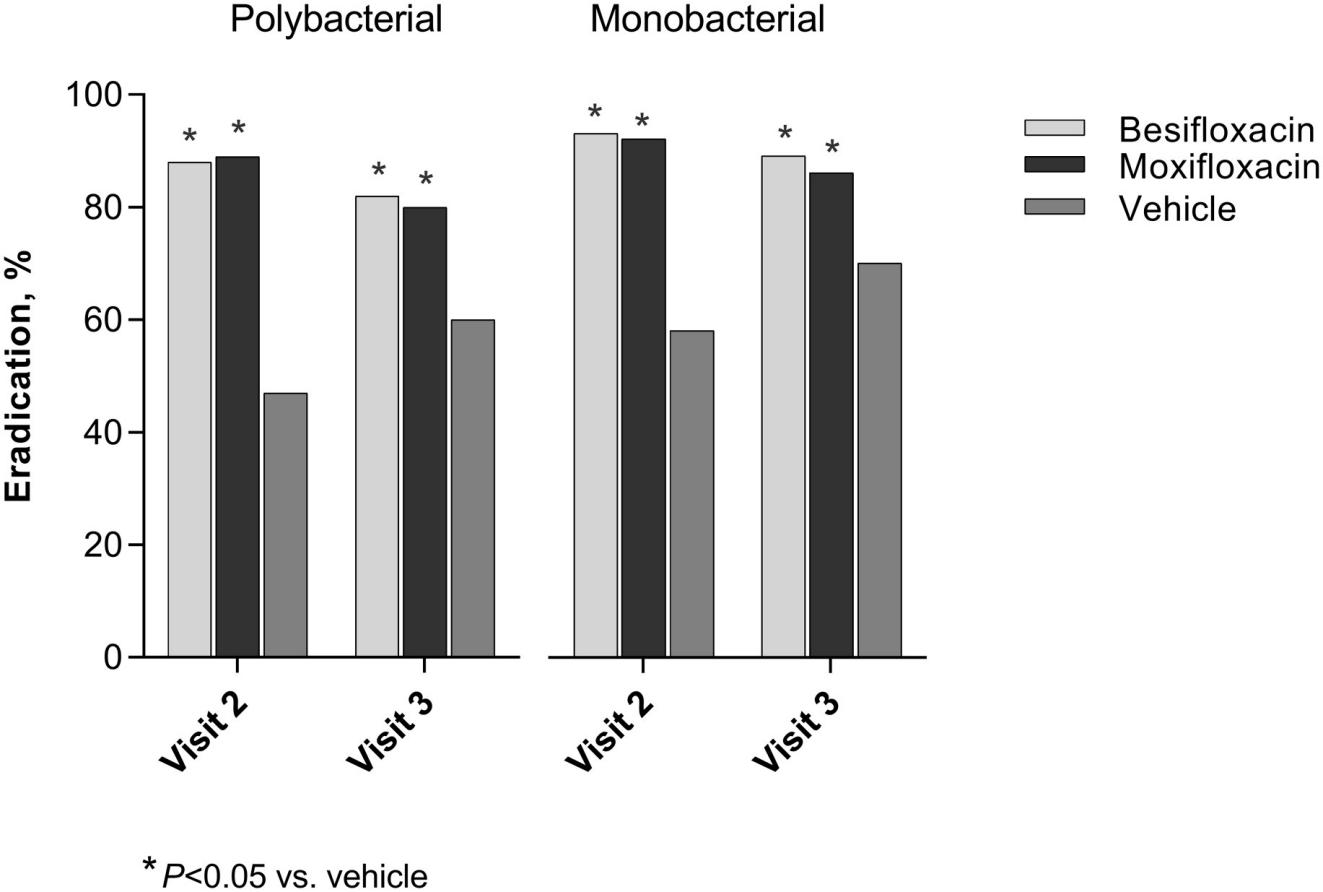
Microbial eradication of polybacterial and monobacterial conjunctivitis with besifloxacin and moxifloxacin.

Analysis of pooled study data showed that both subjects with poly- and mono-bacterial conjunctivitis infections presented high microbial eradication rates at both follow-up visits when treated with besifloxacin ophthalmic suspension 0.6%, which, in all cases, were significantly better than the corresponding rates for subjects treated with vehicle (Fig 4). Among subjects with polybacterial conjunctivitis, the respective eradication rates for besifloxacin and for vehicle were 88% (81/92) and 47% (14/30) at visit 2 (*P*<0.001) and 82% (75/92) and 60% (18/30) at visit 3 (*P* = 0.016). Eradication rates with moxifloxacin ophthalmic suspension 0.5% (included in the active comparator study [32]) were similar to those of besifloxacin, with 89% (49/55) and 80% (44/55) of polybacterial infections eradicated at visits 2 and 3, respectively, and were also better than with vehicle (*P*<0.001 at visit 2, and at visit 3 [*P* = 0.047]; Fig 4).

Among subjects with monobacterial conjunctivitis, the respective microbial eradication rates for besifloxacin and vehicle were 93% (390/419) and 58% (128/219) at visit 2 (*P*<0.001), and 89% (374/419) and 70% (154/219) at visit 3 (*P*<0.001). Eradication rates with moxifloxacin were again similar to those with besifloxacin, with 92% (207/226) and 86% (194/226) of monobacterial infections eradicated at visit 2 and visit 3, respectively (*P*<0.001 vs vehicle at both visits; Fig 4).

Analyses and corresponding p-values can be found in S1 File.

## Discussion

The current study was undertaken to characterize polybacterial infections at baseline across three previously conducted bacterial conjunctivitis studies in comparison to monobacterial infections. We also evaluated the antibacterial efficacy of besifloxacin in such infections. Of the more than 1,000 subjects with culture-confirmed conjunctivitis enrolled across these studies, approximately one in five were infected with more than one bacterial pathogen at baseline. Notably, we applied prespecified species-specific threshold criteria when identifying causative pathogens to avoid attributing conjunctivitis infection to commensal bacteria. This likely accounts for the lower rate of polymicrobial conjunctivitis observed in this analysis vs those reported in three previous studies evaluating polymicrobial conjunctivitis that utilized molecular biology techniques and found rates of 41%–70% [41–43]. The most common dominant infecting bacterium in polybacterial infections in our study was *H*. *influenzae* followed by *S*. *aureus*, with the latter also the most common secondary coinfecting species. *S*. *epidermis* was the second most common secondary coinfecting bacterial species in polybacterial infections. Consistent with other studies [10,18], few polybacterial infections featured combinations of Gram-negative bacteria only.

Significant differences were found between poly- and monobacterial conjunctivitis infections with respect to the prevalence of specific infecting bacteria. While *H*. *influenzae* was the most common dominant infecting species in polybacterial infections as well as the most common infecting species in monobacterial infections, it occurred somewhat less frequently as such in polybacterial infections. *S*. *pneumoniae* was identified as the dominant infecting species about half as often in subjects with polybacterial conjunctivitis versus being the causative infecting species in monobacterial conjunctivitis. Interestingly, a significantly greater proportion of *S*. *pneumoniae* from polybacterial infections were found to be encapsulated, whether considering *S*. *pneumoniae* isolates characterized as dominant in the infection (27.3% encapsulated) or all *S*. *pneumoniae* isolates from polybacterial infections regardless of rank order in the infection (41.4% encapsulated), compared to *S*. *pneumoniae* from monobacterial infections (4.8%). These findings suggest encapsulation may provide a selective advantage to *S*. *pneumoniae* in the presence of other coinfecting bacterial species. The finding of a greater proportion of *S*. *mitis*/mitis group isolates and *S*. *oralis* isolates as the dominant infecting species in polybacterial infections is also intriguing. The alpha hemolytic *S*. *mitis* and other variants in the *S*. *mitis* group is typically considered a commensal bacterium that primarily inhabits the oral cavity [44,45] but can be opportunistic [45–47]. Virulence genes harbored by these species may contribute to evasion of immune defenses, colonization, and adhesion [45]. Further, it has been reported that *S*. *mitis* may exchange resistance genes with neighboring microbes [48]. The appearance of *S*. *mitis*/mitis group as the predominant infecting species in 7.9% of polybacterial infections in the current study suggests there may be polymicrobial interactions at play that render this species opportunistic under certain conditions. *S*. *maltophilia* is a Gram-negative organism that is emerging as a global opportunistic pathogen and is often multidrug resistant [49]. While identified infrequently in this pooled analysis of studies in bacterial conjunctivitis, isolates of *S*. *maltophilia* occurred more often in polybacterial infections than in monobacterial infections and its presence should be monitored in future conjunctivitis studies.

In recent years, and as reflected above, there has been increased recognition of antibiotic resistance in the pathogenesis of ocular infections including in bacterial conjunctivitis [50–53]. Haas et al previously reported on the prevalence of in vitro antibacterial resistance at baseline among bacterial isolates in this same pooled dataset [27]. In the current analysis of MIC data by baseline infection type, few, if any, differences were found for isolates from polybacterial infections compared to isolates from monobacterial infections. Thus, MIC data for poly- and monobacterial infections were also similar to those previously reported for the overall dataset, with high rates of azithromycin resistance observed, and with newer fluoroquinolones (besifloxacin, moxifloxacin, gatifloxacin) generally having greater potency compared to older fluoroquinolones (ciprofloxacin, levofloxacin, ofloxacin) against Gram-positive isolates [27]. This was also the case when evaluating MIC data specific to MRSA and MRSE isolates from poly- vs monobacterial infections: not only did our analysis fail to show a difference in the prevalence of methicillin resistance among staphylococcal isolates by infection type, but MICs did not appear to differ between the methicillin-resistance staphylococcal isolates by infection type. It follows that, in our analysis, in vitro drug resistance did not contribute to coinfection in polybacterial infections.

Multiple species in polymicrobial infections may act collectively to facilitate disease progression. Synergistic interactions may facilitate polymicrobial infection (eg, by one species creating favorable conditions for another species to colonize and/or grow) and enhance virulence, for example by modifying expression of virulence genes [54–57]. To date, the literature on interactions in mixed-pathogen infections has focused largely on biofilms, which can protect infectious bacteria against host innate immune responses [7,58], as well as increase antimicrobial resistance and enhance virulence/persistence. While the contribution of biofilms to acute bacterial conjunctivitis is unclear, studies have shown high proportions of *S*. *aureus* and *S*. *epidermis* isolates from conjunctivitis infections capable of forming biofilms, with lower proportions of isolates from healthy conjunctiva exhibiting this capacity [59–63]. Biofilm-forming ability among conjunctivitis isolates has also been associated with diminished antibiotic susceptibility [59,60]. In this context, it is notable that the one exception to our analysis of differences in MICs by infection type was that higher MICs were observed among Centers for Disease Control (CDC) coryneform spp from polybacterial infections, which were often found in conjunction with staphylococci, compared with those from monobacterial infections. The finding of a higher rate of concurrent viral infection among subjects with polybacterial compared to monobacterial infections is also interesting. It is well known that in systemic infections, for instance in respiratory pneumonia, infection with influenza virus predisposes patients to secondary bacterial infections, often with poorer clinical outcomes [64]. In these infections the initial viral infection renders the host immune response inadequate to defend against secondary bacterial infections. It follows that similar mixed-pathogen/host interactions may occur on the eye.

In conjunctivitis infections where a mixture of bacterial pathogens may be present, the broad-spectrum, potent activity of besifloxacin should be more than ample to eradicate these organisms. Besifloxacin is a fluoroquinolone with structural modifications intended to increase its inhibition of bacterial DNA gyrase and topoisomerase IV [65] and has been reported to be highly bactericidal with potent activity against a wide range of Gram-positive and Gram-negative organisms, including drug-resistant pathogens [25–28,30,66]. In this post-hoc analysis, the *in vitro* activity of besifloxacin was similar to or exceeded that of comparator fluoroquinolone agents against most isolates regardless of infection type. Besifloxacin’s activity was especially notable for Gram-positive organisms where it demonstrated an MIC_90_ 16-fold lower than that of ciprofloxacin, regardless of infection type. In conjunction with its *in vitro* potency, clinical microbiological eradication rates with besifloxacin were similarly robust against both polybacterial and monobacterial infections attesting to the efficacy of this chlorinated fluoroquinolone necessary for empiric use. While eradications rates with besifloxacin were also similar to those with moxifloxacin (evaluated in the active comparator study), the lower MICs of besifloxacin against methicillin-resistant staphylococci compared to that of moxifloxacin, reported here and elsewhere [25,27] bears consideration and may result in differential treatment outcomes in populations of subjects with either poly- or monobacterial conjunctivitis infections due to more resistant strains.

There were several limitations to this post-hoc analysis. Although a robust number of subjects with polybacterial conjunctivitis infections were identified across the studies, sample sizes for unique pairings/combinations of bacterial pathogens were too small to make further inferences. Further, it is not known whether application of fold-differences in CFU/mL over the prespecified threshold criteria is an appropriate way to rank order infecting species in polybacterial infections. Systemic breakpoints for oxacillin were used to interpret MICs for staphylococci as to methicillin sensitivity/resistance, which may be of limited value for determining clinical methicillin resistance in bacterial conjunctivitis. Finally, additional studies will be needed to evaluate the presence of toxins and resistance genes in polybacterial versus monobacterial conjunctivitis infections.

## Conclusions

In studies evaluating besifloxacin ophthalmic suspension 0.6% in the treatment of bacterial conjunctivitis, one in five bacterial conjunctivitis infections were polybacterial, with significant differences in etiology compared to monobacterial infections and underscoring the need for empiric therapy of conjunctivitis to consider mixed pathogen infections. Treatment of subjects with polybacterial conjunctivitis infections with besifloxacin ophthalmic suspension 0.6% resulted in high eradication rates, which were comparable to those for subjects with monobacterial infections.

## Supporting information

S1 File Polybacterial conjunctivitis infections. (RTF)

S2 File ARVO 2019 polymicrobial poster 3APR19. (PDF)
