# Supplementary material for: Characterization of baseline polybacterial versus monobacterial infections in three randomized controlled bacterial conjunctivitis trials and microbial outcomes with besifloxacin ophthalmic suspension 0.6%
Source: PLoS One. 2020 Aug 25;15(8):e0237603. doi: 10.1371/journal.pone.0237603 (PMC7447040; doi:10.1371/journal.pone.0237603)
Supplement: S1 File — (RTF) [file pone.0237603.s001.rtf]

1.	Gender Comparison
Gender Distribution by Study, Treatment, and Infection Type

All 3 Studies Combined	
	besivance	vigamox	vehicle	All	
	sex	sex	sex	sex	
	M	F	M	F	M	F	M	F	
	n	%	n	%	n	%	n	%	n	%	n	%	n	%	n	%	
Monomicrobial	173	41.29	246	58.71	106	46.90	120	53.10	97	44.29	122	55.71	376	43.52	488	56.48	
Polymicrobial	35	38.04	57	61.96	33	60.00	22	40.00	9	30.00	21	70.00	77	43.50	100	56.50	


Gender Distribution by Study, Treatment, and Infection Type

The FREQ Procedure	

Table 1 of blinfect by sex	
Controlling for PROJID=373	
blinfect	sex	
Frequency
Row Pct	0	1	Total	
Monomicrobial	44
44.00	56
56.00	100
	
Polymicrobial	8
44.44	10
55.56	18
	
Total	52	66	118	
Frequency Missing = 151	


Table 2 of blinfect by sex	
Controlling for PROJID=433	
blinfect	sex	
Frequency
Row Pct	0	1	Total	
Monomicrobial	139
41.25	198
58.75	337
	
Polymicrobial	14
26.42	39
73.58	53
	
Total	153	237	390	
Frequency Missing = 567	


Table 3 of blinfect by sex	
Controlling for PROJID=434	
blinfect	sex	
Frequency
Row Pct	0	1	Total	
Monomicrobial	193
45.20	234
54.80	427
	
Polymicrobial	55
51.89	51
48.11	106
	
Total	248	285	533	
Frequency Missing = 628	


Gender Distribution by Study, Treatment, and Infection Type

The FREQ Procedure	

Summary Statistics for blinfect by sex
Controlling for PROJID	

Cochran-Mantel-Haenszel Statistics (Based on Table Scores)	
Statistic	Alternative Hypothesis	DF	Value	Prob	
1	Nonzero Correlation	1	0.0304	0.8615	
2	Row Mean Scores Differ	1	0.0304	0.8615	
3	General Association	1	0.0304	0.8615	


Common Odds Ratio and Relative Risks	
Statistic	Method	Value	95% Confidence Limits	
Odds Ratio	Mantel-Haenszel	1.0298	0.7412	1.4308	
	Logit	1.0116	0.7231	1.4152	
					
Relative Risk (Column 1)	Mantel-Haenszel	1.0166	0.8440	1.2246	
	Logit	0.9646	0.8042	1.1571	
					
Relative Risk (Column 2)	Mantel-Haenszel	0.9874	0.8582	1.1360	
	Logit	0.9346	0.8176	1.0684	


Breslow-Day Test for
Homogeneity of the Odds Ratios	
Chi-Square	5.7443	
DF	2	
Pr > ChiSq	0.0566	


Sample Size = 1041
Frequency Missing = 1346	

WARNING: 56% of the data are missing.	


2. Age Comparison

Age Summary by Study, Treatment, and Infection Type

All 3 Studies Combined	
	besivance	vigamox	vehicle	All	
	Age	Age	Age	Age	
	n	Mean	S.D.	Min.	Max.	n	Mean	S.D.	Min.	Max.	n	Mean	S.D.	Min.	Max.	n	Mean	S.D.	Min.	Max.	
Monomicrobial	419	27.30	24.06	1	92	226	37.00	27.27	0	100	219	26.93	24.04	1	87	864	29.74	25.28	0	100	
Polymicrobial	92	30.47	28.16	1	98	55	43.47	29.14	1	88	30	20.97	24.93	1	81	177	32.90	28.90	1	98	


Age Summary by Study, Treatment, and Infection Type

The GLM Procedure	

Class Level Information	
Class	Levels	Values	
PROJID	3	373 433 434	
blinfect	2	Monomicrobial Polymicrobial	


Number of Observations Read	1041	
Number of Observations Used	1041	


Age Summary by Study, Treatment, and Infection Type

The GLM Procedure	

Dependent Variable: AGE   Age	

Source	DF	Sum of Squares	Mean Square	F Value	Pr > F	
Model	3	32304.9452	10768.3151	16.73	<.0001	
Error	1037	667506.7090	643.6902			
Corrected Total	1040	699811.6542				


R-Square	Coeff Var	Root MSE	AGE Mean	
0.046162	83.78942	25.37105	30.27954	


Source	DF	Type I SS	Mean Square	F Value	Pr > F	
PROJID	2	31691.69854	15845.84927	24.62	<.0001	
blinfect	1	613.24663	613.24663	0.95	0.3293	


Source	DF	Type III SS	Mean Square	F Value	Pr > F	
PROJID	2	30842.41882	15421.20941	23.96	<.0001	
blinfect	1	613.24663	613.24663	0.95	0.3293	


Age Summary by Study, Treatment, and Infection Type

The GLM Procedure	
Least Squares Means	

PROJID	AGE LSMEAN	Standard
Error	Pr > |t|	LSMEAN Number	
373	32.3816656	2.4469107	<.0001	1	
433	24.0257779	1.4950205	<.0001	2	
434	35.7110175	1.2678849	<.0001	3	


Least Squares Means for effect PROJID
Pr > |t| for H0: LSMean(i)=LSMean(j)

Dependent Variable: AGE	
i/j	1	2	3	
1		0.0018	0.1977	
2	0.0018		<.0001	
3	0.1977	<.0001		


	To ensure overall protection level, only probabilities associated with pre-planned comparisons should be used.	


blinfect	AGE LSMEAN	Standard
Error	H0:LSMEAN=0	H0:LSMean1=LSMean2	
			Pr > |t|	Pr > |t|	
Monomicrobial	29.6813178	1.0198235	<.0001	0.3293	
Polymicrobial	31.7309895	2.0042811	<.0001		


Age Summary by Study, Treatment, and Infection Type

The TTEST Procedure	

Variable:  AGE  (Age)	

blinfect	Method	N	Mean	Std Dev	Std Err	Minimum	Maximum	
Monomicrobial		864	29.7431	25.2757	0.8599	0	100.0	
Polymicrobial		177	32.8983	28.9017	2.1724	1.0000	98.0000	
Diff (1-2)	Pooled		-3.1552	25.9256	2.1390			
Diff (1-2)	Satterthwaite		-3.1552		2.3364			


blinfect	Method	Mean	95% CL Mean	Std Dev	95% CL Std Dev	
Monomicrobial		29.7431	28.0553	31.4308	25.2757	24.1375	26.5273	
Polymicrobial		32.8983	28.6110	37.1856	28.9017	26.1719	32.2722	
Diff (1-2)	Pooled	-3.1552	-7.3525	1.0420	25.9256	24.8573	27.0905	
Diff (1-2)	Satterthwaite	-3.1552	-7.7582	1.4477				


Method	Variances	DF	t Value	Pr > |t|	
Pooled	Equal	1039	-1.48	0.1405	
Satterthwaite	Unequal	234.3	-1.35	0.1782	


Equality of Variances	
Method	Num DF	Den DF	F Value	Pr > F	
Folded F	176	863	1.31	0.0171	


Age Summary by Study, Treatment, and Infection Type

The TTEST Procedure	

Variable:  AGE  (Age)	

Protocol ID=373

blinfect	Method	N	Mean	Std Dev	Std Err	Minimum	Maximum	
Monomicrobial		100	30.3400	22.4905	2.2491	1.0000	89.0000	
Polymicrobial		18	39.0556	29.3688	6.9223	2.0000	81.0000	
Diff (1-2)	Pooled		-8.7156	23.6241	6.0487			
Diff (1-2)	Satterthwaite		-8.7156		7.2785			


blinfect	Method	Mean	95% CL Mean	Std Dev	95% CL Std Dev	
Monomicrobial		30.3400	25.8774	34.8026	22.4905	19.7468	26.1267	
Polymicrobial		39.0556	24.4508	53.6603	29.3688	22.0380	44.0281	
Diff (1-2)	Pooled	-8.7156	-20.6957	3.2646	23.6241	20.9360	27.1104	
Diff (1-2)	Satterthwaite	-8.7156	-23.8636	6.4325				


Method	Variances	DF	t Value	Pr > |t|	
Pooled	Equal	116	-1.44	0.1523	
Satterthwaite	Unequal	20.739	-1.20	0.2446	


Equality of Variances	
Method	Num DF	Den DF	F Value	Pr > F	
Folded F	17	99	1.71	0.1081	


Age Summary by Study, Treatment, and Infection Type

The TTEST Procedure	

Variable:  AGE  (Age)	

Protocol ID=433

blinfect	Method	N	Mean	Std Dev	Std Err	Minimum	Maximum	
Monomicrobial		337	23.4807	22.8864	1.2467	1.0000	87.0000	
Polymicrobial		53	22.0000	25.3445	3.4813	1.0000	98.0000	
Diff (1-2)	Pooled		1.4807	23.2309	3.4328			
Diff (1-2)	Satterthwaite		1.4807		3.6978			


blinfect	Method	Mean	95% CL Mean	Std Dev	95% CL Std Dev	
Monomicrobial		23.4807	21.0284	25.9330	22.8864	21.2791	24.7583	
Polymicrobial		22.0000	15.0142	28.9858	25.3445	21.2730	31.3582	
Diff (1-2)	Pooled	1.4807	-5.2685	8.2299	23.2309	21.7051	24.9892	
Diff (1-2)	Satterthwaite	1.4807	-5.9022	8.8636				


Method	Variances	DF	t Value	Pr > |t|	
Pooled	Equal	388	0.43	0.6665	
Satterthwaite	Unequal	66.024	0.40	0.6901	


Equality of Variances	
Method	Num DF	Den DF	F Value	Pr > F	
Folded F	52	336	1.23	0.2974	


Age Summary by Study, Treatment, and Infection Type

The TTEST Procedure	

Variable:  AGE  (Age)	

Protocol ID=434

blinfect	Method	N	Mean	Std Dev	Std Err	Minimum	Maximum	
Monomicrobial		427	34.5457	26.6329	1.2889	0	100.0	
Polymicrobial		106	37.3019	29.2634	2.8423	1.0000	88.0000	
Diff (1-2)	Pooled		-2.7562	27.1732	2.9488			
Diff (1-2)	Satterthwaite		-2.7562		3.1209			


blinfect	Method	Mean	95% CL Mean	Std Dev	95% CL Std Dev	
Monomicrobial		34.5457	32.0124	37.0790	26.6329	24.9584	28.5500	
Polymicrobial		37.3019	31.6661	42.9377	29.2634	25.7843	33.8364	
Diff (1-2)	Pooled	-2.7562	-8.5489	3.0364	27.1732	25.6327	28.9123	
Diff (1-2)	Satterthwaite	-2.7562	-8.9224	3.4100				


Method	Variances	DF	t Value	Pr > |t|	
Pooled	Equal	531	-0.93	0.3504	
Satterthwaite	Unequal	151.05	-0.88	0.3786	


Equality of Variances	
Method	Num DF	Den DF	F Value	Pr > F	
Folded F	105	426	1.21	0.2022	


3. Species Incidence
Incidence of Organisms by Infection Type

	Monomicrobial	Polymicrobial	
	n	% (subj)	% (isol)	n	% (subj)	% (isol)	
Organism	1	0.1	0.1	.	.	.	
ABIOTROPHIA DEFECTIVA							
ACHROMOBACTER XYLOSOXIDANS	1	0.1	0.1	2	1.1	0.5	
ACINETOBACTER CALCOACETICUS	.	.	.	3	1.7	0.8	
ACINETOBACTER JOHNSONII	.	.	.	1	0.6	0.3	
ACINETOBACTER SPECIES	1	0.1	0.1	.	.	.	
AEROCOCCUS VIRIDANS	3	0.3	0.3	5	2.8	1.3	
BACILLUS SPECIES	.	.	.	1	0.6	0.3	
BREVIBACTERIUM CASEI	1	0.1	0.1	.	.	.	
BREVIBACTERIUM SPECIES	5	0.6	0.6	3	1.7	0.8	
BREVUNDIMONAS VESICULARIS	.	.	.	1	0.6	0.3	
CDC CORYNEFORM GROUP G	13	1.5	1.5	10	5.6	2.6	
CDC CORYNEFORM GROUP I1	.	.	.	1	0.6	0.3	
CITROBACTER KOSERI	.	.	.	1	0.6	0.3	
COAGULASE NEGATIVE STAPHYLOCOCCI	.	.	.	2	1.1	0.5	
CORYNEBACTERIUM AFERMENTANS	.	.	.	1	0.6	0.3	
CORYNEBACTERIUM AMYCOLATUM	.	.	.	1	0.6	0.3	
CORYNEBACTERIUM ARGENTORATENSE	.	.	.	2	1.1	0.5	
CORYNEBACTERIUM AURIS	.	.	.	1	0.6	0.3	
CORYNEBACTERIUM JEIKEIUM	2	0.2	0.2	.	.	.	
CORYNEBACTERIUM MACGINLEYI	1	0.1	0.1	5	2.8	1.3	
CORYNEBACTERIUM MINUTISSIMUM	1	0.1	0.1	1	0.6	0.3	
CORYNEBACTERIUM PROPINQUUM	2	0.2	0.2	4	2.3	1.0	
CORYNEBACTERIUM PSEUDODIPHTHERITICUM	3	0.3	0.3	4	2.3	1.0	
CORYNEBACTERIUM SPECIES	2	0.2	0.2	.	.	.	
CORYNEBACTERIUM STRIATUM	3	0.3	0.3	5	2.8	1.3	
CORYNEBACTERIUM UREALYTICUM	1	0.1	0.1	.	.	.	
EIKENELLA CORRODENS	.	.	.	1	0.6	0.3	
ENTEROBACTER CLOACAE	1	0.1	0.1	.	.	.	
ENTEROBACTER SAKAZAKII	.	.	.	1	0.6	0.3	
ENTEROCOCCUS FAECALIS	.	.	.	3	1.7	0.8	
FERMENTATIVE GRAM-NEGATIVE ROD	.	.	.	1	0.6	0.3	
GEMELLA MORBILLORUM	1	0.1	0.1	.	.	.	
GEMELLA SPECIES	.	.	.	2	1.1	0.5	
GRANULICATELLA ADIACENS	2	0.2	0.2	1	0.6	0.3	
HAEMOPHILUS INFLUENZAE	288	33.3	33.3	51	28.8	13.2	
HAEMOPHILUS PARAINFLUENZAE	1	0.1	0.1	3	1.7	0.8	
KINGELLA DENITRIFICANS	.	.	.	1	0.6	0.3	
KLEBSIELLA OXYTOCA	1	0.1	0.1	.	.	.	
KLEBSIELLA OZAENAE	.	.	.	1	0.6	0.3	
KOCURIA KRISTINAE	1	0.1	0.1	.	.	.	
LEMINORELLA SPECIES	1	0.1	0.1	.	.	.	
MICROCOCCUS SPECIES	1	0.1	0.1	1	0.6	0.3	
MORAXELLA CATARRHALIS	5	0.6	0.6	7	4.0	1.8	
MORAXELLA LACUNATA	8	0.9	0.9	1	0.6	0.3	
MORAXELLA NONLIQUEFACIENS	.	.	.	1	0.6	0.3	
MORAXELLA SPECIES	1	0.1	0.1	.	.	.	
MORGANELLA MORGANII	.	.	.	2	1.1	0.5	
NEISSERIA GONORRHOEAE	2	0.2	0.2	.	.	.	
NEISSERIA MENINGITIDIS	3	0.3	0.3	.	.	.	
NEISSERIA SICCA	.	.	.	1	0.6	0.3	
NEISSERIA SUBFLAVA	.	.	.	1	0.6	0.3	
NON-FERMENTATIVE GRAM-NEGATIVE ROD	1	0.1	0.1	2	1.1	0.5	
PASTEURELLA MULTOCIDA	1	0.1	0.1	.	.	.	
PROTEUS MIRABILIS	3	0.3	0.3	2	1.1	0.5	
PSEUDOMONAS AERUGINOSA	3	0.3	0.3	5	2.8	1.3	
ROTHIA MUCILAGINOSA	1	0.1	0.1	3	1.7	0.8	
SERRATIA MARCESCENS	4	0.5	0.5	5	2.8	1.3	
STAPHYLOCOCCUS AUREUS	110	12.7	12.7	73	41.2	18.9	
STAPHYLOCOCCUS CAPITIS	2	0.2	0.2	3	1.7	0.8	
STAPHYLOCOCCUS CAPRAE	3	0.3	0.3	1	0.6	0.3	
STAPHYLOCOCCUS EPIDERMIDIS	54	6.2	6.2	41	23.2	10.6	
STAPHYLOCOCCUS HAEMOLYTICUS	1	0.1	0.1	2	1.1	0.5	
STAPHYLOCOCCUS HOMINIS	6	0.7	0.7	2	1.1	0.5	
STAPHYLOCOCCUS INTERMEDIUS	1	0.1	0.1	.	.	.	
STAPHYLOCOCCUS LUGDUNENSIS	2	0.2	0.2	4	2.3	1.0	
STAPHYLOCOCCUS WARNERI	4	0.5	0.5	1	0.6	0.3	
STAPHYLOCOCCUS XYLOSUS	1	0.1	0.1	.	.	.	
STENOTROPHOMONAS MALTOPHILIA	2	0.2	0.2	7	4.0	1.8	
STREPTOCOCCUS AGALACTIAE	.	.	.	1	0.6	0.3	
STREPTOCOCCUS ANGINOSUS	1	0.1	0.1	.	.	.	
STREPTOCOCCUS ANGINOSUS GROUP	.	.	.	1	0.6	0.3	
STREPTOCOCCUS DYSGALACTIAE	1	0.1	0.1	1	0.6	0.3	
STREPTOCOCCUS MILLERI GROUP	.	.	.	1	0.6	0.3	
STREPTOCOCCUS MITIS	4	0.5	0.5	10	5.6	2.6	
STREPTOCOCCUS MITIS GROUP	17	2.0	2.0	24	13.6	6.2	
STREPTOCOCCUS ORALIS	3	0.3	0.3	14	7.9	3.6	
STREPTOCOCCUS PARASANGUINIS	1	0.1	0.1	1	0.6	0.3	
STREPTOCOCCUS PNEUMONIAE	272	31.5	31.5	29	16.4	7.5	
STREPTOCOCCUS PYOGENES	2	0.2	0.2	3	1.7	0.8	
STREPTOCOCCUS SALIVARIUS	.	.	.	7	4.0	1.8	
STREPTOCOCCUS SANGUINIS	1	0.1	0.1	2	1.1	0.5	
STREPTOCOCCUS SPECIES	5	0.6	0.6	7	4.0	1.8	
STREPTOCOCCUS THERMOPHILUS	1	0.1	0.1	.	.	.	
VIRIDANS STREPTOCOCCUS	1	0.1	0.1	2	1.1	0.5	


4. Eradication Comparison

Eradication of BL Species by Visit, Study, Treatment, and Infection Type

Study Visit=V2

All 3 Studies Combined	
	besivance	vigamox	vehicle	All	
	eradicate	eradicate	eradicate	eradicate	
	No	Yes	No	Yes	No	Yes	No	Yes	
	n	%	n	%	n	%	n	%	n	%	n	%	n	%	n	%	
Monomicrobial	29	6.92	390	93.08	19	8.41	207	91.59	91	41.55	128	58.45	139	16.09	725	83.91	
Polymicrobial	11	11.96	81	88.04	6	10.91	49	89.09	16	53.33	14	46.67	33	18.64	144	81.36	


Eradication of BL Species by Visit, Study, Treatment, and Infection Type

Study Visit=V3

All 3 Studies Combined	
	besivance	vigamox	vehicle	All	
	eradicate	eradicate	eradicate	eradicate	
	No	Yes	No	Yes	No	Yes	No	Yes	
	n	%	n	%	n	%	n	%	n	%	n	%	n	%	n	%	
Monomicrobial	45	10.74	374	89.26	32	14.16	194	85.84	65	29.68	154	70.32	142	16.44	722	83.56	
Polymicrobial	17	18.48	75	81.52	11	20.00	44	80.00	12	40.00	18	60.00	40	22.60	137	77.40	


Eradication of BL Species by Visit, Study, Treatment, and Infection Type

Eradication Rates Across Studies by Visit and Treatment

The FREQ Procedure	

visittyp=V2 treatmnt=besivance

Table of PROJID by eradicate	
PROJID(Protocol ID)	eradicate	
Frequency
Row Pct	0	1	Total	
373	6
10.00	54
90.00	60
	
433	17
8.54	182
91.46	199
	
434	17
6.75	235
93.25	252
	
Total	40	471	511	


Statistics for Table of PROJID by eradicate	

Statistic	DF	Value	Prob	
Chi-Square	2	0.9421	0.6244	
Likelihood Ratio Chi-Square	2	0.9276	0.6289	
Mantel-Haenszel Chi-Square	1	0.9363	0.3332	
Phi Coefficient		0.0429		
Contingency Coefficient		0.0429		
Cramer's V		0.0429		

Sample Size = 511	


Eradication of BL Species by Visit, Study, Treatment, and Infection Type

Eradication Rates Across Studies by Visit and Treatment

The FREQ Procedure	

visittyp=V2 treatmnt=vigamox

Table of PROJID by eradicate	
PROJID(Protocol ID)	eradicate	
Frequency
Row Pct	0	1	Total	
434	25
8.90	256
91.10	281
	
Total	25	256	281	


Eradication of BL Species by Visit, Study, Treatment, and Infection Type

Eradication Rates Across Studies by Visit and Treatment

The FREQ Procedure	

visittyp=V2 treatmnt=vehicle

Table of PROJID by eradicate	
PROJID(Protocol ID)	eradicate	
Frequency
Row Pct	0	1	Total	
373	30
51.72	28
48.28	58
	
433	77
40.31	114
59.69	191
	
Total	107	142	249	


Statistics for Table of PROJID by eradicate	

Statistic	DF	Value	Prob	
Chi-Square	1	2.3635	0.1242	
Likelihood Ratio Chi-Square	1	2.3460	0.1256	
Continuity Adj. Chi-Square	1	1.9209	0.1658	
Mantel-Haenszel Chi-Square	1	2.3540	0.1250	
Phi Coefficient		0.0974		
Contingency Coefficient		0.0970		
Cramer's V		0.0974		


Fisher's Exact Test	
Cell (1,1) Frequency (F)	30	
Left-sided Pr <= F	0.9540	
Right-sided Pr >= F	0.0832	
		
Table Probability (P)	0.0372	
Two-sided Pr <= P	0.1325	

Sample Size = 249	


Eradication of BL Species by Visit, Study, Treatment, and Infection Type

Eradication Rates Across Studies by Visit and Treatment

The FREQ Procedure	

visittyp=V3 treatmnt=besivance

Table of PROJID by eradicate	
PROJID(Protocol ID)	eradicate	
Frequency
Row Pct	0	1	Total	
373	7
11.67	53
88.33	60
	
433	23
11.56	176
88.44	199
	
434	32
12.70	220
87.30	252
	
Total	62	449	511	


Statistics for Table of PROJID by eradicate	

Statistic	DF	Value	Prob	
Chi-Square	2	0.1496	0.9279	
Likelihood Ratio Chi-Square	2	0.1496	0.9279	
Mantel-Haenszel Chi-Square	1	0.1134	0.7363	
Phi Coefficient		0.0171		
Contingency Coefficient		0.0171		
Cramer's V		0.0171		

Sample Size = 511	


Eradication of BL Species by Visit, Study, Treatment, and Infection Type

Eradication Rates Across Studies by Visit and Treatment

The FREQ Procedure	

visittyp=V3 treatmnt=vigamox

Table of PROJID by eradicate	
PROJID(Protocol ID)	eradicate	
Frequency
Row Pct	0	1	Total	
434	43
15.30	238
84.70	281
	
Total	43	238	281	


Eradication of BL Species by Visit, Study, Treatment, and Infection Type

Eradication Rates Across Studies by Visit and Treatment

The FREQ Procedure	

visittyp=V3 treatmnt=vehicle

Table of PROJID by eradicate	
PROJID(Protocol ID)	eradicate	
Frequency
Row Pct	0	1	Total	
373	23
39.66	35
60.34	58
	
433	54
28.27	137
71.73	191
	
Total	77	172	249	


Statistics for Table of PROJID by eradicate	

Statistic	DF	Value	Prob	
Chi-Square	1	2.6987	0.1004	
Likelihood Ratio Chi-Square	1	2.6197	0.1055	
Continuity Adj. Chi-Square	1	2.1921	0.1387	
Mantel-Haenszel Chi-Square	1	2.6878	0.1011	
Phi Coefficient		0.1041		
Contingency Coefficient		0.1035		
Cramer's V		0.1041		


Fisher's Exact Test	
Cell (1,1) Frequency (F)	23	
Left-sided Pr <= F	0.9629	
Right-sided Pr >= F	0.0708	
		
Table Probability (P)	0.0337	
Two-sided Pr <= P	0.1073	

Sample Size = 249	


Eradication of BL Species by Visit, Study, Treatment, and Infection Type

Besi vs Vehicle Comparison of Eradication Rates by Infection Type and Visit

The FREQ Procedure	

blinfect=Monomicrobial visittyp=V2

Table of treatmnt by eradicate	
treatmnt	eradicate	
Frequency
Row Pct	0	1	Total	
besivance	29
6.92	390
93.08	419
	
vehicle	91
41.55	128
58.45	219
	
Total	120	518	638	


Statistics for Table of treatmnt by eradicate	

Statistic	DF	Value	Prob	
Chi-Square	1	112.9548	<.0001	
Likelihood Ratio Chi-Square	1	108.7118	<.0001	
Continuity Adj. Chi-Square	1	110.6984	<.0001	
Mantel-Haenszel Chi-Square	1	112.7777	<.0001	
Phi Coefficient		-0.4208		
Contingency Coefficient		0.3878		
Cramer's V		-0.4208		


Fisher's Exact Test	
Cell (1,1) Frequency (F)	29	
Left-sided Pr <= F	<.0001	
Right-sided Pr >= F	1.0000	
		
Table Probability (P)	<.0001	
Two-sided Pr <= P	<.0001	

Sample Size = 638	


Eradication of BL Species by Visit, Study, Treatment, and Infection Type

Besi vs Vehicle Comparison of Eradication Rates by Infection Type and Visit

The FREQ Procedure	

blinfect=Monomicrobial visittyp=V3

Table of treatmnt by eradicate	
treatmnt	eradicate	
Frequency
Row Pct	0	1	Total	
besivance	45
10.74	374
89.26	419
	
vehicle	65
29.68	154
70.32	219
	
Total	110	528	638	


Statistics for Table of treatmnt by eradicate	

Statistic	DF	Value	Prob	
Chi-Square	1	36.1606	<.0001	
Likelihood Ratio Chi-Square	1	34.4138	<.0001	
Continuity Adj. Chi-Square	1	34.8454	<.0001	
Mantel-Haenszel Chi-Square	1	36.1039	<.0001	
Phi Coefficient		-0.2381		
Contingency Coefficient		0.2316		
Cramer's V		-0.2381		


Fisher's Exact Test	
Cell (1,1) Frequency (F)	45	
Left-sided Pr <= F	<.0001	
Right-sided Pr >= F	1.0000	
		
Table Probability (P)	<.0001	
Two-sided Pr <= P	<.0001	

Sample Size = 638	


Eradication of BL Species by Visit, Study, Treatment, and Infection Type

Besi vs Vehicle Comparison of Eradication Rates by Infection Type and Visit

The FREQ Procedure	

blinfect=Polymicrobial visittyp=V2

Table of treatmnt by eradicate	
treatmnt	eradicate	
Frequency
Row Pct	0	1	Total	
besivance	11
11.96	81
88.04	92
	
vehicle	16
53.33	14
46.67	30
	
Total	27	95	122	


Statistics for Table of treatmnt by eradicate	

Statistic	DF	Value	Prob	
Chi-Square	1	22.4748	<.0001	
Likelihood Ratio Chi-Square	1	20.1593	<.0001	
Continuity Adj. Chi-Square	1	20.1379	<.0001	
Mantel-Haenszel Chi-Square	1	22.2906	<.0001	
Phi Coefficient		-0.4292		
Contingency Coefficient		0.3944		
Cramer's V		-0.4292		


Fisher's Exact Test	
Cell (1,1) Frequency (F)	11	
Left-sided Pr <= F	<.0001	
Right-sided Pr >= F	1.0000	
		
Table Probability (P)	<.0001	
Two-sided Pr <= P	<.0001	

Sample Size = 122	


Eradication of BL Species by Visit, Study, Treatment, and Infection Type

Besi vs Vehicle Comparison of Eradication Rates by Infection Type and Visit

The FREQ Procedure	

blinfect=Polymicrobial visittyp=V3

Table of treatmnt by eradicate	
treatmnt	eradicate	
Frequency
Row Pct	0	1	Total	
besivance	17
18.48	75
81.52	92
	
vehicle	12
40.00	18
60.00	30
	
Total	29	93	122	


Statistics for Table of treatmnt by eradicate	

Statistic	DF	Value	Prob	
Chi-Square	1	5.7829	0.0162	
Likelihood Ratio Chi-Square	1	5.3771	0.0204	
Continuity Adj. Chi-Square	1	4.6561	0.0309	
Mantel-Haenszel Chi-Square	1	5.7355	0.0166	
Phi Coefficient		-0.2177		
Contingency Coefficient		0.2127		
Cramer's V		-0.2177		


Fisher's Exact Test	
Cell (1,1) Frequency (F)	17	
Left-sided Pr <= F	0.0178	
Right-sided Pr >= F	0.9949	
		
Table Probability (P)	0.0126	
Two-sided Pr <= P	0.0251	

Sample Size = 122	


Eradication of BL Species by Visit, Study, Treatment, and Infection Type

Besi vs Vigamox Comparison of Eradication Rates by Infection Type and Visit

The FREQ Procedure	

blinfect=Monomicrobial visittyp=V2

Table of treatmnt by eradicate	
treatmnt	eradicate	
Frequency
Row Pct	0	1	Total	
besivance	29
6.92	390
93.08	419
	
vigamox	19
8.41	207
91.59	226
	
Total	48	597	645	


Statistics for Table of treatmnt by eradicate	

Statistic	DF	Value	Prob	
Chi-Square	1	0.4706	0.4927	
Likelihood Ratio Chi-Square	1	0.4628	0.4963	
Continuity Adj. Chi-Square	1	0.2796	0.5970	
Mantel-Haenszel Chi-Square	1	0.4698	0.4931	
Phi Coefficient		-0.0270		
Contingency Coefficient		0.0270		
Cramer's V		-0.0270		


Fisher's Exact Test	
Cell (1,1) Frequency (F)	29	
Left-sided Pr <= F	0.2953	
Right-sided Pr >= F	0.8015	
		
Table Probability (P)	0.0968	
Two-sided Pr <= P	0.5304	

Sample Size = 645	


Eradication of BL Species by Visit, Study, Treatment, and Infection Type

Besi vs Vigamox Comparison of Eradication Rates by Infection Type and Visit

The FREQ Procedure	

blinfect=Monomicrobial visittyp=V3

Table of treatmnt by eradicate	
treatmnt	eradicate	
Frequency
Row Pct	0	1	Total	
besivance	45
10.74	374
89.26	419
	
vigamox	32
14.16	194
85.84	226
	
Total	77	568	645	


Statistics for Table of treatmnt by eradicate	

Statistic	DF	Value	Prob	
Chi-Square	1	1.6329	0.2013	
Likelihood Ratio Chi-Square	1	1.5983	0.2061	
Continuity Adj. Chi-Square	1	1.3238	0.2499	
Mantel-Haenszel Chi-Square	1	1.6303	0.2017	
Phi Coefficient		-0.0503		
Contingency Coefficient		0.0503		
Cramer's V		-0.0503		


Fisher's Exact Test	
Cell (1,1) Frequency (F)	45	
Left-sided Pr <= F	0.1255	
Right-sided Pr >= F	0.9188	
		
Table Probability (P)	0.0443	
Two-sided Pr <= P	0.2054	

Sample Size = 645	


Eradication of BL Species by Visit, Study, Treatment, and Infection Type

Besi vs Vigamox Comparison of Eradication Rates by Infection Type and Visit

The FREQ Procedure	

blinfect=Polymicrobial visittyp=V2

Table of treatmnt by eradicate	
treatmnt	eradicate	
Frequency
Row Pct	0	1	Total	
besivance	11
11.96	81
88.04	92
	
vigamox	6
10.91	49
89.09	55
	
Total	17	130	147	


Statistics for Table of treatmnt by eradicate	

Statistic	DF	Value	Prob	
Chi-Square	1	0.0369	0.8476	
Likelihood Ratio Chi-Square	1	0.0372	0.8471	
Continuity Adj. Chi-Square	1	0.0000	1.0000	
Mantel-Haenszel Chi-Square	1	0.0367	0.8481	
Phi Coefficient		0.0158		
Contingency Coefficient		0.0158		
Cramer's V		0.0158		


Fisher's Exact Test	
Cell (1,1) Frequency (F)	11	
Left-sided Pr <= F	0.6710	
Right-sided Pr >= F	0.5365	
		
Table Probability (P)	0.2075	
Two-sided Pr <= P	1.0000	

Sample Size = 147	


Eradication of BL Species by Visit, Study, Treatment, and Infection Type

Besi vs Vigamox Comparison of Eradication Rates by Infection Type and Visit

The FREQ Procedure	

blinfect=Polymicrobial visittyp=V3

Table of treatmnt by eradicate	
treatmnt	eradicate	
Frequency
Row Pct	0	1	Total	
besivance	17
18.48	75
81.52	92
	
vigamox	11
20.00	44
80.00	55
	
Total	28	119	147	


Statistics for Table of treatmnt by eradicate	

Statistic	DF	Value	Prob	
Chi-Square	1	0.0517	0.8201	
Likelihood Ratio Chi-Square	1	0.0514	0.8206	
Continuity Adj. Chi-Square	1	0.0001	0.9918	
Mantel-Haenszel Chi-Square	1	0.0513	0.8207	
Phi Coefficient		-0.0188		
Contingency Coefficient		0.0187		
Cramer's V		-0.0188		


Fisher's Exact Test	
Cell (1,1) Frequency (F)	17	
Left-sided Pr <= F	0.4913	
Right-sided Pr >= F	0.6747	
		
Table Probability (P)	0.1660	
Two-sided Pr <= P	0.8310	

Sample Size = 147	


Eradication of BL Species by Visit, Study, Treatment, and Infection Type

Vigamox vs Vehicle Comparison of Eradication Rates by Infection Type and Visit

The FREQ Procedure	

blinfect=Monomicrobial visittyp=V2

Table of treatmnt by eradicate	
treatmnt	eradicate	
Frequency
Row Pct	0	1	Total	
vigamox	19
8.41	207
91.59	226
	
vehicle	91
41.55	128
58.45	219
	
Total	110	335	445	


Statistics for Table of treatmnt by eradicate	

Statistic	DF	Value	Prob	
Chi-Square	1	65.6633	<.0001	
Likelihood Ratio Chi-Square	1	69.9482	<.0001	
Continuity Adj. Chi-Square	1	63.8942	<.0001	
Mantel-Haenszel Chi-Square	1	65.5157	<.0001	
Phi Coefficient		-0.3841		
Contingency Coefficient		0.3586		
Cramer's V		-0.3841		


Fisher's Exact Test	
Cell (1,1) Frequency (F)	19	
Left-sided Pr <= F	<.0001	
Right-sided Pr >= F	1.0000	
		
Table Probability (P)	<.0001	
Two-sided Pr <= P	<.0001	

Sample Size = 445	


Eradication of BL Species by Visit, Study, Treatment, and Infection Type

Vigamox vs Vehicle Comparison of Eradication Rates by Infection Type and Visit

The FREQ Procedure	

blinfect=Monomicrobial visittyp=V3

Table of treatmnt by eradicate	
treatmnt	eradicate	
Frequency
Row Pct	0	1	Total	
vigamox	32
14.16	194
85.84	226
	
vehicle	65
29.68	154
70.32	219
	
Total	97	348	445	


Statistics for Table of treatmnt by eradicate	

Statistic	DF	Value	Prob	
Chi-Square	1	15.7183	<.0001	
Likelihood Ratio Chi-Square	1	15.9518	<.0001	
Continuity Adj. Chi-Square	1	14.8209	0.0001	
Mantel-Haenszel Chi-Square	1	15.6830	<.0001	
Phi Coefficient		-0.1879		
Contingency Coefficient		0.1847		
Cramer's V		-0.1879		


Fisher's Exact Test	
Cell (1,1) Frequency (F)	32	
Left-sided Pr <= F	<.0001	
Right-sided Pr >= F	1.0000	
		
Table Probability (P)	<.0001	
Two-sided Pr <= P	<.0001	

Sample Size = 445	


Eradication of BL Species by Visit, Study, Treatment, and Infection Type

Vigamox vs Vehicle Comparison of Eradication Rates by Infection Type and Visit

The FREQ Procedure	

blinfect=Polymicrobial visittyp=V2

Table of treatmnt by eradicate	
treatmnt	eradicate	
Frequency
Row Pct	0	1	Total	
vigamox	6
10.91	49
89.09	55
	
vehicle	16
53.33	14
46.67	30
	
Total	22	63	85	


Statistics for Table of treatmnt by eradicate	

Statistic	DF	Value	Prob	
Chi-Square	1	18.2124	<.0001	
Likelihood Ratio Chi-Square	1	17.8473	<.0001	
Continuity Adj. Chi-Square	1	16.0681	<.0001	
Mantel-Haenszel Chi-Square	1	17.9982	<.0001	
Phi Coefficient		-0.4629		
Contingency Coefficient		0.4201		
Cramer's V		-0.4629		


Fisher's Exact Test	
Cell (1,1) Frequency (F)	6	
Left-sided Pr <= F	<.0001	
Right-sided Pr >= F	1.0000	
		
Table Probability (P)	<.0001	
Two-sided Pr <= P	<.0001	

Sample Size = 85	


Eradication of BL Species by Visit, Study, Treatment, and Infection Type

Vigamox vs Vehicle Comparison of Eradication Rates by Infection Type and Visit

The FREQ Procedure	

blinfect=Polymicrobial visittyp=V3

Table of treatmnt by eradicate	
treatmnt	eradicate	
Frequency
Row Pct	0	1	Total	
vigamox	11
20.00	44
80.00	55
	
vehicle	12
40.00	18
60.00	30
	
Total	23	62	85	


Statistics for Table of treatmnt by eradicate	

Statistic	DF	Value	Prob	
Chi-Square	1	3.9341	0.0473	
Likelihood Ratio Chi-Square	1	3.8283	0.0504	
Continuity Adj. Chi-Square	1	2.9860	0.0840	
Mantel-Haenszel Chi-Square	1	3.8878	0.0486	
Phi Coefficient		-0.2151		
Contingency Coefficient		0.2103		
Cramer's V		-0.2151		


Fisher's Exact Test	
Cell (1,1) Frequency (F)	11	
Left-sided Pr <= F	0.0433	
Right-sided Pr >= F	0.9866	
		
Table Probability (P)	0.0299	
Two-sided Pr <= P	0.0726	

Sample Size = 85	


5. Dominant Organism Incidence
Dominant Organism by Infection Type

Dominant Organism	Monomicrobial	Polymicrobial	p-value	
	n	% (subj)	n	% (subj)		
HAEMOPHILUS INFLUENZAE	288	33.3	45	25.4	0.042	
STAPHYLOCOCCUS AUREUS	110	12.7	26	14.7	NS	
STAPHYLOCOCCUS EPIDERMIDIS	54	6.2	6	3.4	NS	
STENOTROPHOMONAS MALTOPHILIA	2	0.2	6	3.4	<0.001	
STREPTOCOCCUS MITIS / MITIS GROUP	21	2.4	14	7.9	<0.001	
STREPTOCOCCUS ORALIS	3	0.3	5	2.8	0.005	
STREPTOCOCCUS PNEUMONIAE	272	31.5	22	12.4	<0.001	
