# Supplementary material for: Characterization of baseline polybacterial versus monobacterial infections in three randomized controlled bacterial conjunctivitis trials and microbial outcomes with besifloxacin ophthalmic suspension 0.6%
Source: PLoS One. 2020 Aug 25;15(8):e0237603. doi: 10.1371/journal.pone.0237603 (PMC7447040; doi:10.1371/journal.pone.0237603)
Supplement: S2 File — (PDF) [file pone.0237603.s002.pdf]

Poster # 245 - B0364

BAUSCH + LOMB

Incidence of Polybacterial Infections in Three Bacterial Conjunctivitis Studies and Outcomes with Besifloxacin Ophthalmic Suspension 0.6%

Contact information:
Joseph M. Blondeau M.Sc., Ph.D
Royal University Hospital,
Saskatoon Health Region and
University of Saskatchewan
Phone: 306-655-6943
E mail:
Joseph.Blondeau@saskhealthauthority.ca

Joseph M. Blondeau,[1] Christine M. Sanfilippo,[2] Heleen H. DeCory[2]

[1]Clinical Microbiology, Royal University Hospital, Saskatoon, SK, Canada; [2]Pharmaceutical Medical Affairs, Bausch + Lomb, Rochester, NY, USA

Background

- Advances in diagnostics have led to a growing recognition of the polymicrobial nature of many infections[1] including topical ocular infections.[2]
- Interactions between pathogenic and commensal bacteria and/or viruses contributes to disease progression and can impact treatment outcomes. Studies of polymicrobial infections have documented alterations in virulence factors, biofilm formation, and antibiotic resistance or tolerance.[1] While much research has focused on the pathogenesis of systemic polymicrobial infections, little is known to date specific to ocular polymicrobial infections.
- Besifloxacin is a fluoroquinolone antibacterial indicated for the topical treatment of bacterial conjunctivitis, with molecular modifications to increase its potency relative to other fluoroquinolones (**Fig. 1**).[3]
  - In addition to the N-cyclopropyl group, besifloxacin has an 8-chloro substituent that improves inhibition of DNA gyrase and topoisomerase IV.
  - Besifloxacin is highly bactericidal with broad spectrum activity against a range of bacterial pathogens, including drug-resistant pathogens.[4-7]
- To gain insight into the contribution of polymicrobial infections to ocular disease, we assessed the incidence of polybacterial infections at baseline in clinical pivotal studies of besifloxacin ophthalmic suspension 0.6% (Besivance®, Bausch & Lomb Incorporated) in the treatment of bacterial conjunctivitis.[8-10] We also report on bacterial eradication outcomes with besifloxacin in subjects with polybacterial conjunctivitis.

Figure 1. Structure of besifloxacin

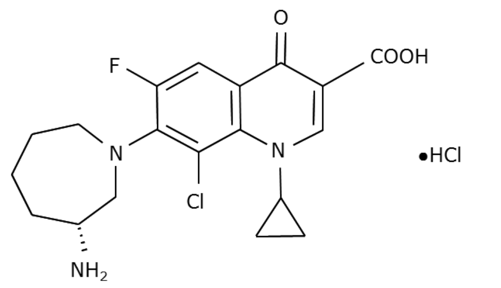

Methods

Data from three multicenter, randomized, double-masked clinical studies (two vehicle-controlled [NCT000622908, NCT00347932] and one active-controlled [NCT00348348]) evaluating the clinical safety and efficacy of besifloxacin ophthalmic suspension 0.6% were pooled.

Clinical study designs, described previously,[8-10] were similar across the studies. Subjects were aged ≥1 year, with a diagnosis of bacterial conjunctivitis including purulent conjunctival discharge and redness in at least one eye. Conjunctival swabs were taken at baseline and two follow-up visits (Visit 2 [Days 4 or 5], and Visit 3 [Days 8 or 9]) following treatment initiation (besifloxacin ophthalmic suspension 0.6%, vehicle, or moxifloxacin ophthalmic solution 0.5%, each instilled TID for 5 days). The same central laboratory was used to identify bacterial species in culture swabs.

Subjects with culture-confirmed polybacterial conjunctivitis at baseline were identified across the three studies, and baseline culture characteristics and clinical bacterial eradication outcome data were extracted for analysis. To avoid attribution of conjunctivitis to commensal (i.e. normal) microflora, isolates were classified as causative only if their colony count (in CFU/mL) equated or exceeded species-specific pre-specified threshold criteria (see listing[11]). The fold-increase in colony count over threshold was used to rank order the contribution of each causative species to the baseline infection.

Minimum inhibitory concentrations (MICs) for besifloxacin and comparator agents (including other flouroquinolones) against baseline isolates deemed causative in polybacterial infections were also extracted and summarized. These were previously determined by broth microdilution according to CLSI guidelines[12] and interpreted based on CLSI systemic breakpoints in effect during study conduct.

Where indicated differences in proportions were evaluated using Chi-square analysis.

Species-Specific Pre-Specified Threshold Criteria:

Group I 1 CFU/mL
- *Streptococcus pyogenes*
- *Streptococcus pneumoniae*
- all Gram-negative bacteria (except *Moraxella catarrhalis*), including: *Achromobacter*, *Acinetobacter*, *Chryseobacterium*, *Citrobacter*, *Eikenella*, *Enterobacter*, *Escherichia*, *Haemophilus*, *Klebsiella*, *Kingella*, *Leminorella*, *Methylobacterium*, *Moraxella* (except *M. catarrhalis*), *Morganella*, *Neisseria*, *Ochrobactrum*, *Pantoea*, *Pasteurella*, *Proteus*, *Pseudomonas*, *Ralstonia*, *Rhizobium*, *Serratia*, *Sphingomonas*, *Stenotrophomonas* (aka. *Xanthomonas*)

Group II 10 CFU/mL
- *Staphylococcus aureus*
- *Streptococcus* (except *S. pyogenes* and *S. pneumoniae*) and related organisms: *Abiotrophia*, *Aerococcus viridans* (aka *Gaffkya homari* or *Pediococcus homari*), *Enterococcus*, *Gemella*, *Granulicatella*, and *Leuconostoc*
- *Moraxella catarrhalis*

Group III 100 CFU/mL
- *Staphylococcus* (except *S. aureus*)
- *Micrococcus* and related organisms: *Kocuria* and *Rothia* (aka *Stomatococcus*)
- *Bacillus*

Group IV 1000 CFU/mL
- *Corynebacterium* (including CDC coryneform group bacteria)
- *Brevibacterium*
- *Oerskovia*
- *Rhodococcus*

Results

- Of 1041 subjects with culture-confirmed bacterial conjunctivitis across the three studies, 17% (117) had polybacterial infections at baseline.
  - 83% were infected with two bacterial species, 15% with three species, and 2% with four species
- Demographics and baseline infection characteristics of subjects with polybacterial infections are shown in **Table 1**.
  - Mean (SD) age was 33 (29) years; and 56% were female
  - 7.9% (14/177) of subjects were co-infected with virus (n=12 adenovirus, n=2 HSV), a proportion significantly greater than that observed in monobacterial infections.
- *Haemophilus influenzae* was the most common dominant causative species in polybacterial infections followed by *Staphylococcus aureus* (**Table 1**, **Fig. 2**) which was also the most common secondary co-infecting species.

Table 1: Subject Demographics and Baseline Infection Characteristics

	Subjects (N=1041)		
	Polybacterial Infections (n=177)	Monobacterial Infections (n=864)	*P*-value*
Mean (SD) age, years	33.1 (28.9)	29.7 (25.3)	--
Gender, female (%)	99 (55.9)	488 (56.5)	NS
Viral co-infection (n,%)	14 (7.9)	14 (1.6)	<.00001
Dominant infecting species			
H. influenzae	46 (26.0)	288 (33.3)	NS
S. aureus	26 (14.7)	110 (12.7)	NS
S. pneumoniae	22 (12.4)	272 (31.5)	<.00001
S. epidermidis	6 (3.4)	54 (6.3)	NS

*Chi-square analysis

Figure 2: Dominant, Secondary, and Tertiary Infecting Species in Polybacterial Conjunctivitis Infections

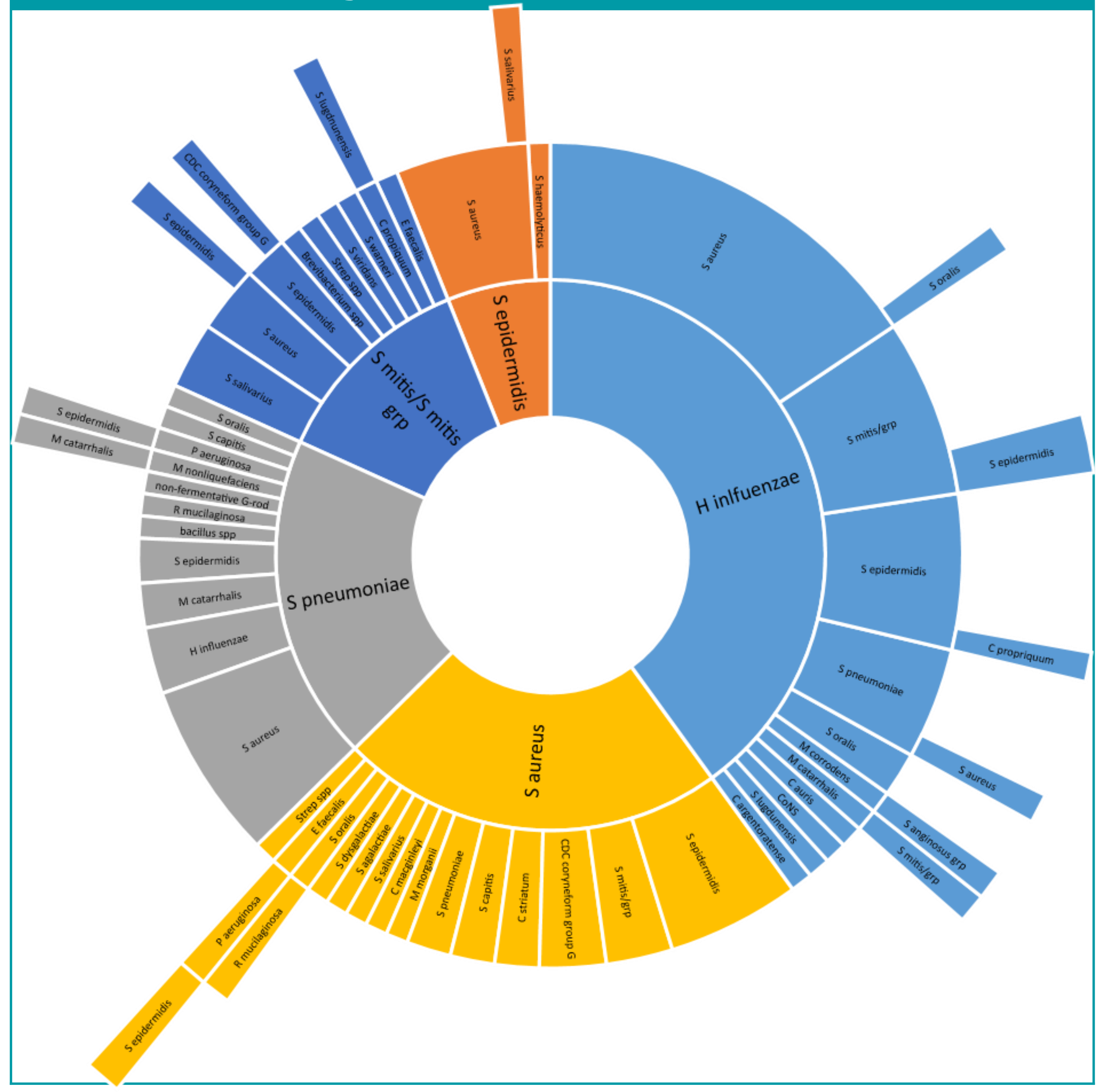

Dominant bacterial species are shown in the inner circle, whereas ancillary secondary and tertiary infecting bacterial species are shown by rank order in the outer rings. Only those polybacterial infections in which the same dominant species was identified in 10 or more patients are presented.

- MIC ranges and MIC_{90}s of besifloxacin for all isolates, Gram-positive isolates, Gram-negative isolates, and for individual species with ≥10 isolates at baseline are shown in **Table 2**, whereas the distribution of MICs for besifloxacin compared to ciprofloxacin against Gram-positive and Gram-negative isolates is shown in **Fig. 3**.

Table 2: MICs of Besifloxacin Against Baseline Isolates from Polybacterial Conjunctivitis Infections

	Range (µg/mL)		MIC_{90} (µg/mL)
	min	max	
All isolates (N=387)	0.015	8.0	0.5
Gram-positive isolates (n=286)	0.015	4.0	0.5
Gram-negative isolates (n=101)	0.015	8.0	2.0
S. aureus (n=73)	0.015	4.0	0.5
H. influenzae (n=51)	0.015	0.5	0.06
S. epidermidis (n=41)	0.03	4.0	0.5
S. mitis/S. mitis group (n=34)	0.03	1.0	0.25
S. pneumoniae (n=29)	0.06	0.25	0.125
S. oralis (n=14)	0.015	0.25	0.25

MIC_{90}: Minimum inhibitory concentration that inhibits 90% of isolates
MIC data is only shown for those baseline species with ≥10 isolates

Figure 3: Distribution of MICs for Besifloxacin and Ciprofloxacin Against Gram-Positive and Gram-Negative Isolates

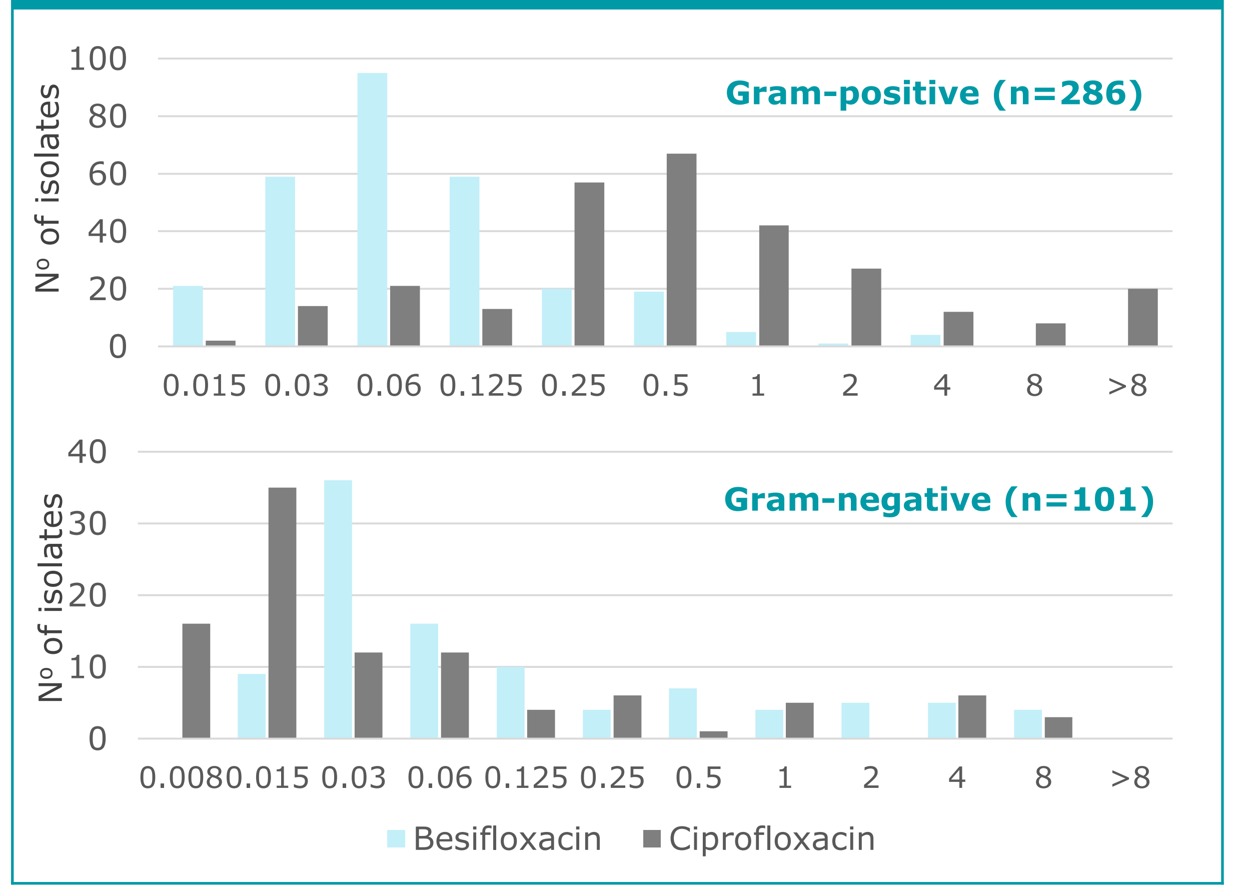

- Treatment of patients with polybacterial conjunctivitis with besifloxacin ophthalmic suspension 0.6% resulted in high bacterial eradication rates at both follow-up visits, and significantly better than with vehicle (**Table 3**).

Table 3: Microbial Eradication of Polybacterial Conjunctivitis Infections

	Besifloxacin % (n/N)	Vehicle % (n/N)	*P*-value*
Visit 2	93% (81/87)	52% (14/27)	<0.00001
Visit 3	90% (75/83)	62% (18/29)	0.000475

*Chi-square analysis
Microbial eradication was defined as the infecting species originally present at or above threshold at baseline is absent in follow-up visit cultures

Conclusions

- Nearly 1 in 5 subjects had polybacterial conjunctivitis at baseline in studies evaluating besifloxacin ophthalmic suspension 0.6% for bacterial conjunctivitis.
- Besifloxacin demonstrated potent *in vitro* activity against isolates from polybacterial infections
- Treatment of subjects with besifloxacin resulted in eradication rates ≥90% for these infections attesting to the broad spectrum activity of this chloro-fluoroquinolone, and required for such infections.

References

1. Short FL, et al. *Trends Microbiol* 2014;22(9):508-16. **2.** Tuft S. *Br J Ophthalmol* 2006;90:257-258. **3.** Ward KH, et al. *J Ocular Phamacol Ther* 2007;23(3): 243-56. **3.** Cambau E, et al. *J Antimicrob Chemother* 2009;63(3): 443-50. **4.** Haas W, et al. *Antimicrob Agents Chemother* 2009;53(8): 3552-60. **5.** Haas W, et al. *J Antimicrob Chemother* 2010; 65(7): 1441-7. **6.** Asbell PA, et al. *JAMA Ophthalmol* 2015;133(12):1445-54. **7.** McDonald MB, et al. *Ophthalmology* 2009; 116(9):1615-23. **8.** Karpecki P, et al. *Clin Ther* 2009;31(3):514-26. **9.** Tepedino ME, et al. *Curr Med Res Opin* 2009; 25(5):1159-69. **10.** Haas W, et al. Poster presented at the 2010 Interational Conference on Ocular Infections, Feb 18-21, West Palm Beach, FL. **11.** Clinical and Laboratory Standards Institute. Methods for Dilution Antimicrobial Susceptibility Tests for Bacteria That Grow Aerobically; Approved Standard-Tenth Edition. CLSI document M7-A10 (2015). Clinical and Laboratory Standards Institute, Wayne, PA.

Presented at the 2019 Annual Meeting of the Association for Research in Vision and Ophthalmology (ARVO) April 28 - May 2, Vancouver, CA.

Disclosure: The studies analysis herein were sponsored by Bausch Health US, LLC. CMS and HHD are employees of Bausch Health US, LLC.; JMB has received research grants from Bausch Health US, LLC.
